# Deregulation of mitochondrial F1FO-ATP synthase via OSCP in Alzheimer’s disease

**DOI:** 10.1038/ncomms11483

**Published:** 2016-05-06

**Authors:** Simon J. Beck, Lan Guo, Aarron Phensy, Jing Tian, Lu Wang, Neha Tandon, Esha Gauba, Lin Lu, Juan M. Pascual, Sven Kroener, Heng Du

**Affiliations:** 1 Department of Biological Sciences, The University of Texas at Dallas, 800W. Campbell Road, Richardson, Texas 75080, USA; 2 School of Behavioral and Brain Sciences, The University of Texas at Dallas, 800W. Campbell Road, Richardson, Texas 75080, USA; 3 Department of Neurology, Shandong Provincial Hospital Affiliated to Shandong University, 324 Jingwu Weiqi Road, Jinan, Shandong 250021, China; 4 Department of Neurology and Neurotherapeutics, The University of Texas Southwestern Medical Center, 5323 Harry Hines Boulevard, Richardson, Texas 75390, USA

## Abstract

F1FO-ATP synthase is critical for mitochondrial functions. The deregulation of this enzyme results in dampened mitochondrial oxidative phosphorylation (OXPHOS) and activated mitochondrial permeability transition (mPT), defects which accompany Alzheimer’s disease (AD). However, the molecular mechanisms that connect F1FO-ATP synthase dysfunction and AD remain unclear. Here, we observe selective loss of the oligomycin sensitivity conferring protein (OSCP) subunit of the F1FO-ATP synthase and the physical interaction of OSCP with amyloid beta (Aβ) in the brains of AD individuals and in an AD mouse model. Changes in OSCP levels are more pronounced in neuronal mitochondria. OSCP loss and its interplay with Aβ disrupt F1FO-ATP synthase, leading to reduced ATP production, elevated oxidative stress and activated mPT. The restoration of OSCP ameliorates Aβ-mediated mouse and human neuronal mitochondrial impairments and the resultant synaptic injury. Therefore, mitochondrial F1FO-ATP synthase dysfunction associated with AD progression could potentially be prevented by OSCP stabilization.

## Abstract (web summary)

F1FO ATP synthase is a critical enzyme for the maintenance of mitochondrial function. Here the authors demonstrate that loss of the F1FO-ATP synthase subunit OSCP and the interaction of OSCP with Aβ peptide in Alzheimer’s disease patients and mouse models lead to F1FO-ATP synthase deregulation and disruption of synaptic mitochondrial function.

## Graphical abstract

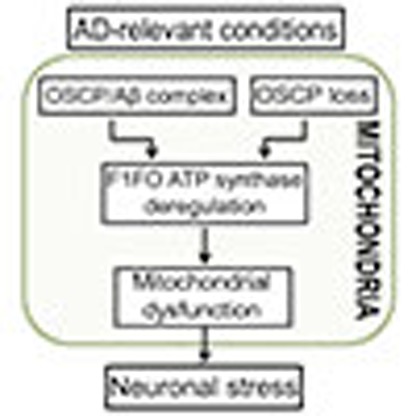

Clinical observations have suggested that mitochondrial dysfunction is among the earliest manifestations of Alzheimer’s disease (AD) and constitutes a hallmark pathological feature of this neurological disorder^1^,^2^. Previous studies have also identified impaired mitochondrial oxidative phosphorylation (OXPHOS) as a feature of mitochondrial defects in AD individuals^2^,^3^ and AD animal models^4^,^5^,^6^. Compromised mitochondrial OXPHOS efficiency results in lowered mitochondrial bioenergetics and exaggerated production of free radicals^7^. Indeed, ATP deficiency and oxidative damage are characteristics of brains from AD patients^2^. Impaired mitochondrial OXPHOS efficiency is closely associated with dysfunction of mitochondrial respiratory enzymes, including mitochondrial complex I to IV, as well as the defect of F1FO-ATP synthase. Previous studies have mostly focused on the dysfunction of mitochondrial complex IV in AD^8^,^9^,^10^. However, this concept has been recently challenged^11^,^12^,^13^. In fact, in addition to mitochondrial respiratory enzyme defects, increasing evidence implicates the dysfunction of mitochondrial F1FO-ATP synthase in AD^14^,^15^,^16^,^17^.

The mitochondrial F1FO-ATP synthase, which includes three components (F1, FO and the peripheral stalk), is a critical mitochondrial OXPHOS enzyme involved in the regulation of mitochondrial ATP production and in the maintenance of the mitochondrial membrane potential^18^,^19^. The F1FO-ATP synthase can both synthesize ATP and degrade ATP when operating in reverse to generate proton backflow, increasing mitochondrial membrane potential when it is critically low^19^,^20^,^21^. In addition to its vital function in mitochondrial OXPHOS, recent studies have shown that this enzyme contributes to the formation of the mitochondrial permeability transition pore (mPTP)^22^,^23^ through the interaction of its oligomycin sensitivity conferring protein (OSCP) subunit with cyclophilin D (CypD), the key regulator of mPTP^22^,^23^,^24^. Extensive formation of mPTP is a severe mitochondrial pathological event that leads to collapsed mitochondrial membrane potential (mΔΨ), decreased mitochondrial OXPHOS capacity, elevated reactive oxygen species (ROS) generation and, eventually, cell death^25^. Indeed, mPTP activation is thought to be a key mechanism of mitochondrial stress in AD and has been proposed to underlie its characteristic synaptic dysfunction and cognitive decline^5^,^26^. Given its role in mitochondrial OXPHOS^18^ and mPTP formation^21^,^22^,^23^, the deregulation of mitochondrial F1FO-ATP synthase may predispose to compromised OXPHOS efficiency and sensitized mPTP formation, which are two hallmark mitochondrial defects in AD. However, to date information on the dysfunction of F1FO-ATP synthase in AD has remained limited. Accordingly, the underlying molecular mechanisms causing the defect of this enzyme in AD remain unresolved.

In this study, we compare the levels of major F1FO-ATP synthase subunits in the brains from AD individuals, mild cognitive impairment (MCI) patients and non-AD control subjects, and find a selective decrease in the levels of OSCP during the progression of AD. We also find that, in a mouse model of AD that overexpresses the human form of amyloid beta (Aβ), the loss of OSCP is more prominent in synaptic mitochondria. In addition to OSCP loss, we also detect a direct physical interaction between OSCP and Aβ in the brains from AD cases, as well as in AD mice. Such OSCP aberrations disrupt F1FO-ATP synthase stability, leading to severe mitochondrial dysfunction and synaptic injury. Further *in vivo* studies show the deleterious impact of F1FO-ATP synthase dysfunction on the development of mitochondrial defects in AD mice. Importantly, the restoration of OSCP ameliorates the Aβ-mediated mitochondrial dysfunction and synaptic injury in mouse or human neurons, further supporting the role of OSCP deregulation in mitochondrial dysfunction under Aβ-rich conditions. Therefore, mitochondrial F1FO-ATP synthase dysfunction that results from OSCP aberrations may constitute a primary AD event that can be prevented by OSCP protection, suggesting OSCP as a potential new therapeutic target for AD.

## Results

### Loss of OSCP in AD subjects and 5xFAD mice

**Figure 1:**
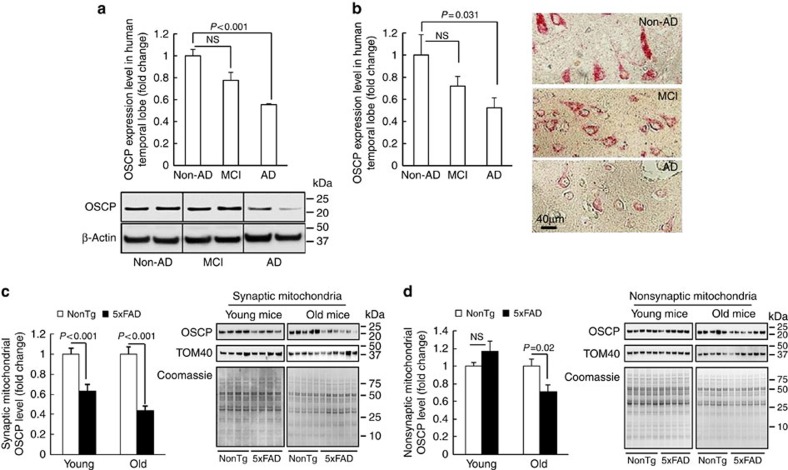
Loss of OSCP in AD subjects and 5xFAD mouse brain mitochondria. (**a**) Densitometric quantification of immunoreactive bands of OSCP in protein extracts from the temporal lobes of non-AD, MCI and AD patients. β-Actin was used to indicate the loading amount of proteins. The lower panel is representative of six non-AD, six MCI and four AD patients. (**b**) Immunohistochemical staining using anti-OSCP on brain sections from human temporal lobes showed substantial reduction of OSCP in neurons. The right panel shows representative images. Scale bar, 40 μm. (**c**,**d**) Densitometric quantification of OSCP immunoreactive bands shows the age-dependent OSCP reduction in synaptic (**c**) and nonsynaptic (**d**) mitochondria from 5xFAD mice. TOM40 was used to show the enrichment of mitochondrial fractions. Coomassie blue staining was employed to indicate the loading amount of mitochondrial proteins. Five nonTg and five 5xFAD mice at 4 months old and 5 nonTg and 6 5xFAD mice at 9 months old were used in the experiments. Error bars represent s.e.m.

To examine changes of F1FO-ATP synthase in AD brains, we used immunoblotting to compare the expression levels of the major subunits of the mitochondrial F1FO-ATP synthase in protein extracts from the temporal lobe of non-AD, MCI, and AD subjects (Supplementary Table 1). Among the F1FO-ATP synthase subunits tested here, OSCP was slightly reduced in MCI brains, while this decrease was exaggerated in AD patients (Fig. 1a). Notably, the expression levels of the other major F1FO-ATP synthase subunits, including a, b, c, α, and β were not significantly changed in either MCI or AD brains (Supplementary Fig. 1a,b), implying that the OSCP loss is not likely a result of F1FO-ATP synthase reduction in AD. Next, we performed histological studies on brain sections from the temporal lobes of AD and control subjects. Our results showed a dramatic reduction of OSCP expression in neurons from the temporal lobe of AD individuals (Fig. 1b). A similar reduction of OSCP also occurred in an AD mouse model (5xFAD mice). To determine whether synaptic mitochondria are more vulnerable to OSCP loss under conditions of elevated Aβ, we separated synaptic mitochondria and nonsynaptic mitochondria from 5xFAD mice at 4 and 9 months old that mimic the amyloidopathy and behavioral changes typical of the early-, and middle to late-stages of AD, respectively^27^. The purity of isolated mitochondria was verified by means of the detection of high abundance of the mitochondrial protein voltage-dependent anion channel (VDAC) without contamination of β-actin or synaptic vesicles (Supplementary Fig. 2). Synaptic mitochondria demonstrated a significant OSCP decrease in young 5xFAD mice, and this OSCP loss was exacerbated with age (Fig. 1c). However, a significant OSCP reduction in nonsynaptic mitochondria was detected only in old 5xFAD mice (Fig. 1d), suggesting that in Aβ-rich environments synaptic mitochondria are more susceptible to OSCP loss. In sharp contrast, the expression levels of a, b and c, as well as α and β subunits remained unaltered in either synaptic or nonsynaptic mitochondria from 5xFAD mice at any tested age (Supplementary Fig. 3a–d), indicating that OSCP expression is selectively suppressed in parallel with the manifestation of AD-like symptoms in 5xFAD mice.

### OSCP loss impacts mitochondrial and synaptic function

**Figure 2:**
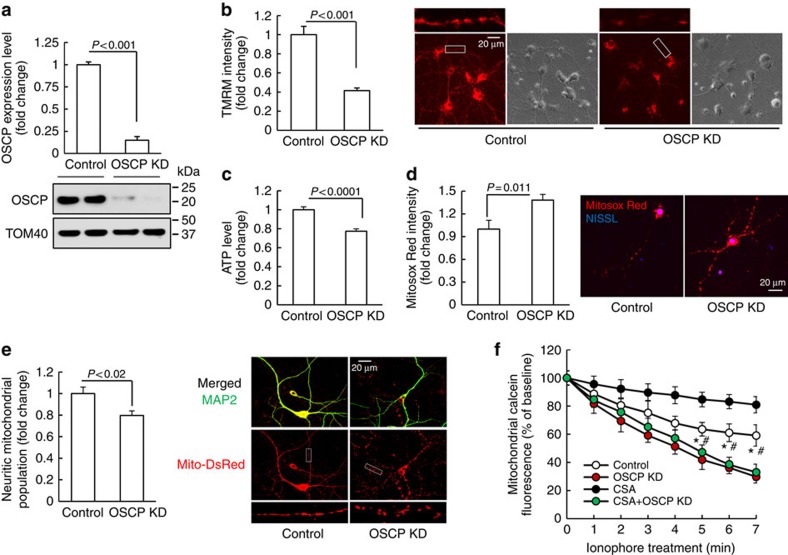
OSCP deficiency impacts neuronal mitochondrial function. (**a**) OSCP expression was downregulated in primary cultured mouse neurons by using lentivirus carrying OSCP shRNA (OSCP KD (OSCP knockdown neurons)) and the control neurons were treated by lentivirus carrying nonTarget shRNA. *n*=6–9 samples. The lower panel shows the representative image of immunoreactive bands of OSCP. TOM40 was used as the loading control. (**b**) OSCP downregulation induced reduction in mitochondrial membrane potential. *n*=36–51 neurons from at least three independent experiments. The right panel shows representatives of TMRM staining and phase contrast images. Scale bar, 20 μm. (**c**) OSCP deficiency also induced decreased neuron ATP production (*n*=13–22 samples from at least three independent experiments). (**d**) OSCP deficiency induced increased mitochondrial superoxide levels (*n*=10–15 neurons from at least three independent experiments). The right panel shows representatives of Mitosox Red staining (red). NISSL (Blue) was used to identify neurons. Scale bar, 20 μm. (**e**) Loss of OSCP induced reduction in neuritic mitochondrial population. The right panel shows representative images of MAP2 (green, dendrites) and Mito-Dsred (red, mitochondria) staining. *n*=27–43 neurons from at least three independent experiments. Scale bar, 20 μm. (**f**) mPTP formation demonstrated by the drop in mitochondrial calcein intensity in the exposure of 2 μM ionophore. CsA was used at 1 μM. *P<0.01 versus OSCP KD neurons in the presence or absence of CsA. ^#^*P*<0.05 versus control neurons treated by CsA. *n*=5–10 independent experiments. Error bars represent s.e.m.

To investigate the impact of OSCP loss on neuronal mitochondrial function we genetically downregulated OSCP expression in primary cultured mouse neurons by using specific OSCP shRNA. Nontarget shRNA was used as control. Quantitative analysis showed substantially decreased OSCP levels in OSCP knockdown neurons (OSCP KD, Fig. 2a). The key functions of mitochondrial F1FO-ATP synthase are to maintain mΔΨ and to produce ATP. To determine the impact of OSCP loss on F1FO-ATP synthase function, we first measured mΔΨ in OSCP knockdown neurons via staining with tetramethylrhodamine methyl ester (TMRM), an indicator of mΔΨ (ref. 5). In comparison with control neurons, OSCP knockdown neurons exhibited significantly decreased intensities of TMRM (Fig. 2b), suggesting a collapsed mΔΨ in response to OSCP loss. Furthermore, in agreement with reduced mΔΨ, ATP generation was markedly reduced in OSCP knockdown neurons (Fig. 2c), suggesting deregulation of mitochondrial OXPHOS efficiency. In view of the close correlation between compromised OXPHOS efficiency and mitochondrial ROS production, we next compared mitochondrial ROS levels by staining with MitoSox Red, a specific fluorescent indicator of mitochondrial superoxide abundance^5^,^6^. Our results showed significantly elevated MitoSox Red intensity in mitochondria from OSCP knockdown neurons (Fig. 2d). In addition, in comparison to the control neurons OSCP downregulated neurons also displayed significantly reduced mitochondrial population in neurites (Fig. 2e).

OSCP deregulation has also been previously linked to mPTP formation^22^,^23^,^24^. To determine whether the loss of OSCP sensitizes mPTP opening, we subjected neurons to calcein AM-cobalt chloride quenching assay^28^. When we exposed the neurons to ionophore, which increases mitochondrial calcium overloading to trigger mPTP formation^29^, OSCP knockdown neurons exhibited larger reductions in mitochondrial calcein intensity than control neurons (Fig. 2f). CypD is a known regulator of mPTP^25^. Inhibiting CypD pharmacologically with cyclosporin A (CsA) substantially suppressed mPTP formation in control neurons; however, mPTP activation associated with OSCP downregulation was not blocked by the addition of CsA (Fig. 2f). These results seem to implicate that mPTP activation induced by loss of OSCP does not rely on the functional status of CypD. This was further supported by unaltered CypD expression levels in OSCP knockdown neurons detected by immunoblotting in parallel with calcein AM-cobalt chloride quenching assay (Supplementary Fig. 4a). Furthermore, by downregulating OSCP in CypD-deficient neurons, we found that mPTP activation resulting from OSCP deficiency was not protected by depletion of CypD (Supplementary Fig. 4b,c), which is in agreement with the abolished effect of CsA treatment (Fig. 2f). Taken together, these data suggest that the loss of OSCP sensitizes mPTP opening regardless of the functional status of CypD.

Because the major function of OSCP is to stabilize the F1FO complex by interacting with F1, we tried to further probe the association between OSCP loss-of-function and mPTP formation in neurons by disrupting the interaction of OSCP with F1. To this end, we downregulated the β-subunit, which is the major F1 component that interacts with OSCP in the integral F1FO-ATP synthase^30^ (Supplementary Fig. 5a). Neurons in which the β-subunit was knockeddown demonstrated significantly suppressed mΔΨ and reduced ATP generation (Supplementary Fig. 5b,c) in patterns similar to those that we observed in OSCP-deficient neurons (Fig. 2b,c). Importantly, β-subunit knockdown neurons displayed substantial mPTP activation (Supplementary Fig. 5d). These results indicated that increased mPTP formation is closely associated with F1FO complex disruption, further highlighting a key role of OSCP loss-of-function in triggering mPTP opening. These findings also support previous reports that increased F1 dissociation from the F1FO complex contributes to mPTP formation^23^,^31^,^32^.

**Figure 3:**
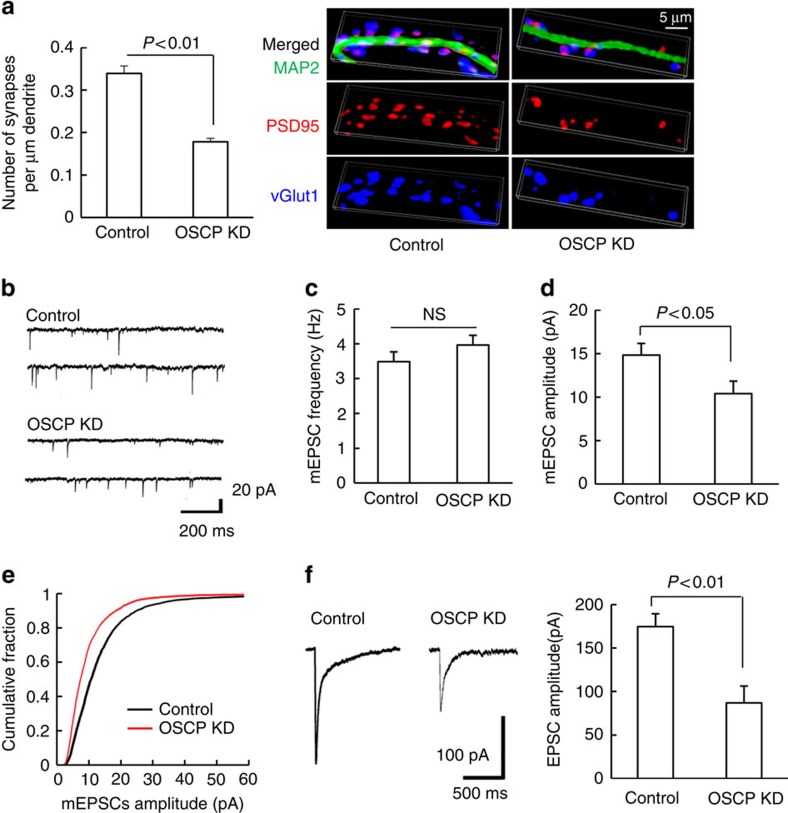
OSCP deficiency impacts synaptic function. (**a**) Loss of OSCP induced a decline in synaptic density. The staining of vGlut1 (blue) and PSD95 (red) were used to identify the pre- and post- synaptic components of synapses, respectively. Neuronal dendrites were identified by staining for MAP2 (green). Scale bar, 5 μm. *n*=27–43 neurons from at least three independent experiments. (**b**–**e**) Loss of OSCP affected mEPSCs. (**b**) Representative traces of mEPSCs in control (upper panel) and OSCP KD neurons (lower panel). Scale bars represent 200 ms and 20 pA. Data were collected from 10 control neurons and 7 OSCP knockdown (OSCP KD) neurons from at least three different cultures. (**c**) Quantitative analysis of mEPSC frequency. (**d**) Quantitative analysis of mEPSC amplitude. (**e**) Analysis of the cumulative fraction of mEPSC amplitude distribution. (**f**) Loss of OSCP reduced the amplitude of EPSCs evoked by puff-application of glutamate. Scale bars represent 500 ms and 100 pA. Data were collected from seven control neurons and eight OSCP KD neurons. The right panel shows quantitative analysis of EPSC amplitude. Error bars represent s.e.m.

Mitochondria play a critical role in maintaining synaptic and neuronal function^26^,^33^. To determine whether loss of OSCP induces synaptic dysfunction, we measured synaptic density by staining for PSD95 and vesicular glutamate transporter 1 (vGlut1). OSCP shRNA-treated neurons showed a significant reduction in synaptic density (Fig. 3a), suggesting a deleterious influence of OSCP loss-associated mitochondrial dysfunction on synaptic function. To directly determine the influence of OSCP loss on synaptic transmission we performed whole-cell voltage-clamp recordings to examine changes in action potential-independent miniature excitatory postsynaptic currents (mEPSCs). The downregulation of OSCP significantly reduced the average mEPSC amplitude in OSCP knockdown neurons in comparison to their nontarget shRNA-treated controls (Fig. 3b,d,e), without significantly altering mEPSC frequency (Fig. 3b,c). Furthermore, direct stimulation of postsynaptic glutamate receptors by ‘puffing’ glutamate onto cultured neurons resulted in currents of significantly smaller amplitude in OSCP knockdown neuron (Fig. 3f). Taken together, these data suggest that OSCP loss impairs synaptic function, which parallels the well-documented synaptic dysfunction, and particularly a loss of postsynaptic function, in the pathology of AD^34^. Energy deprivation, oxidative stress and mPTP formation are hallmarks of mitochondrial defects in AD brains^2^,^5^,^35^,^36^. Synaptic loss and impaired synaptic transmission further characterize AD-related pathological changes^34^,^37^,^38^,^39^. Therefore, our results suggest that reduced OSCP expression is associated with AD-like mitochondrial dysfunction and the resultant synaptic failure.

### Interaction of OSCP with Aβ results in OSCP loss-of-function

**Figure 4:**
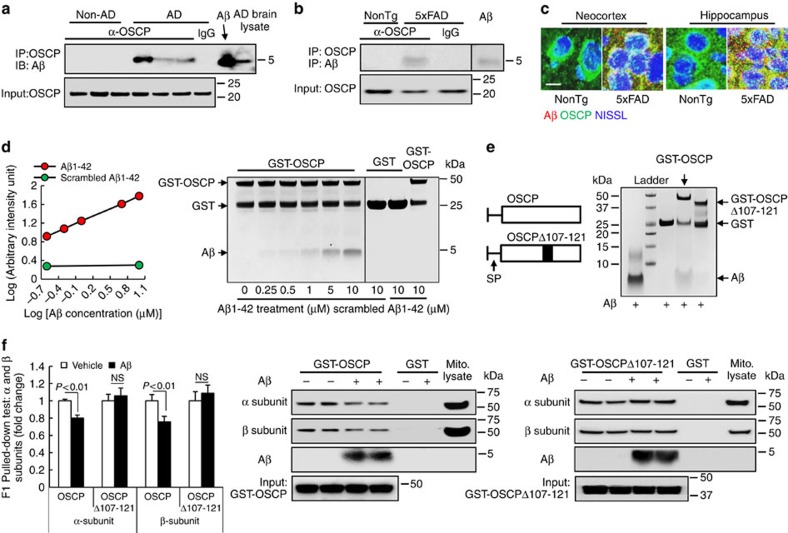
OSCP/Aβ interactions impact OSCP function *in vivo* and *in vitro*. (**a**) Co-immunoprecipitation of OSCP and Aβ in AD patient temporal lobes. Results shown are representatives from three non-AD and three AD patients. Aβ1–42 peptide and AD brain extracts were used as positive controls for Aβ immunoreactive bands. (**b**) Co-immunoprecipitation of OSCP and Aβ in 5xFAD mouse neocortex. Results shown are representatives from three mice in each group. Aβ1–42 peptide was used as a positive control for Aβ immunoreactive bands. (**c**) Colocalization of OSCP (green) and Aβ (red) in neocortex and hippocampus from 5xFAD mice. Neurons were identified by staining for NISSL (blue). Scale bar, 10 μm. (**d**) OSCP and Aβ interaction determined by an *in vitro* pull-down assay. (**e**) Amino-acid residue numbers are given for mature OSCP protein (with known mitochondrial signalling peptide removed). SP is the mitochondrial signalling peptide. Wild-type OSCP and OSCPΔ107–121 were used for pull-down assay. OSCPΔ107–121 displayed lowered capacity to interact with Aβ compared to wild-type OSCP. (**f**) *In vitro* pull-down assay showed that Aβ1–42 suppresses the ability of OSCP, but not OSCPΔ107–121, to bind α- and β-subunits as demonstrated by decreased α- and β-immunoreactive bands. *n*=6–10 samples per group. Error bars represent s.e.m.

Aβ is a key mediator of AD and previous studies have shown that Aβ deposits in AD mitochondria, targeting several mitochondrial proteins^2^,^5^,^35^,^40^. We therefore explored whether OSCP is a binding partner of Aβ in AD brains by co-immunoprecipitating temporal lobe protein extracts using anti-OSCP antibody. We detected OSCP/Aβ interaction in AD brains (Fig. 4a), as well as in MCI brains that showed brain Aβ deposition (Supplementary Fig. 6). In contrast, non-AD brains did not exhibit the OSCP/Aβ complex (Fig. 4a). The OSCP/Aβ complex was not detected in AD brain tissue when the OSCP antibody was replaced by nonimmune IgG (Fig. 4a), validating the specificity of OSCP/Aβ binding. Similarly, the OSCP/Aβ complex was also found in 5xFAD mice by co-immunoprecipitation (Fig. 4b). Further confocal microscopy studies showed extensive colocalization of OSCP and Aβ in neocortex and hippocampus from 5xFAD mice (Fig. 4c), suggesting an interaction between OSCP and Aβ *in vivo*. To determine whether OSCP directly interacts with Aβ we next performed an *in vitro* pull-down assay using glutathione S-transferase (GST)-tagged OSCP as the bait protein. Aβ1–42, but not scrambled Aβ1–42, demonstrated dose-dependent binding with OSCP (Fig. 4d), indicating direct physical interaction of OSCP with Aβ.

Physical binding of Aβ could result in functional changes of its target proteins^2^. Therefore, it is important to determine whether the Aβ interaction influences OSCP function by tethering F1 via binding with α- and β-subunits^41^. To address this question, GST-tagged OSCP was pre-incubated with or without Aβ1–42. After washing off unbound Aβ1–42, we conducted *in vitro* pull-down assays and found that the binding of OSCP to α- or β-subunits was significantly reduced by Aβ1–42, suggesting that the interplay between OSCP and Aβ1–42 disrupts the ability of OSCP to bind to the F1 entity (Fig. 4f). To further evaluate the association between Aβ and impaired OSCP function, we generated deleted forms of OSCP based on the predicted Aβ binding sites on OSCP (Supplementary Fig. 7a–c). We found that the ability of OSCPΔ107–121 to interact with Aβ was significantly reduced (Fig. 4e). Furthermore, OSCPΔ107–121 showed extensive binding with α- or β-subunits regardless of the presence or absence of Aβ1–42 (Fig. 4f). These results further indicate a direct and specific influence of Aβ on OSCP, leading to reduced mitochondrial bioenergetics and activated mPTP formation.

To examine the impact of Aβ on OSCP binding to the F1FO-ATP synthase within mitochondrial membranes we exposed brain mitochondria to Aβ1–42. As predicted, co-immunoprecipitation showed OSCP/Aβ complexes in Aβ-exposed mitochondria, indicating that Aβ1–42 can enter mitochondria and bind to OSCP within the F1FO-ATP synthase (Supplementary Fig. 8a). Next, we examined F1FO-ATP synthase stability by conducting blue-native PAGE (BN-PAGE)^42^,^43^. Mitochondria were exposed to vehicle-, Aβ1–42- or Ca^2+^-treatment. Ca^2+^ is thought to disrupt the F1FO complex through OSCP^32^. Aβ induced a marked increase in F1 dissociation from the F1FO complex, similar to Ca^2+^-treatment, while little OSCP was detected in the free F1 (Supplementary Fig. 8b,c). The identification of F1FO dimer, monomer and F1 by β subunit blots was further validated by using an antibody against F1 (Supplementary Fig. 8d). Together these data suggest that the Aβ-induced F1FO complex instability is at least partly due to a reduction in OSCP’s ability to hold F1 and FO together. As expected, Aβ1–42-treated mitochondria displayed mitochondrial OXPHOS inhibition as evident from the significantly suppressed ATP synthesis (Supplementary Fig. 8e). To dissect the influence of Aβ on mitochondrial OXPHOS enzymes, we measured the enzymatic activities of mitochondrial complexes I through IV and of the F1FO-ATP synthase. F1FO-ATP synthase activity was significantly reduced in exogenous Aβ-treated mitochondria (Supplementary Fig. 8f); while the activities of mitochondrial complexes I to IV remained unaltered (Supplementary Fig. 8g–j), suggesting that F1FO-ATP synthase dysfunction confers Aβ toxicity to mitochondrial OXPHOS under the experimental conditions tested.

### OSCP aberrations and 5xFAD mouse mitochondrial dysfunction

**Figure 5:**
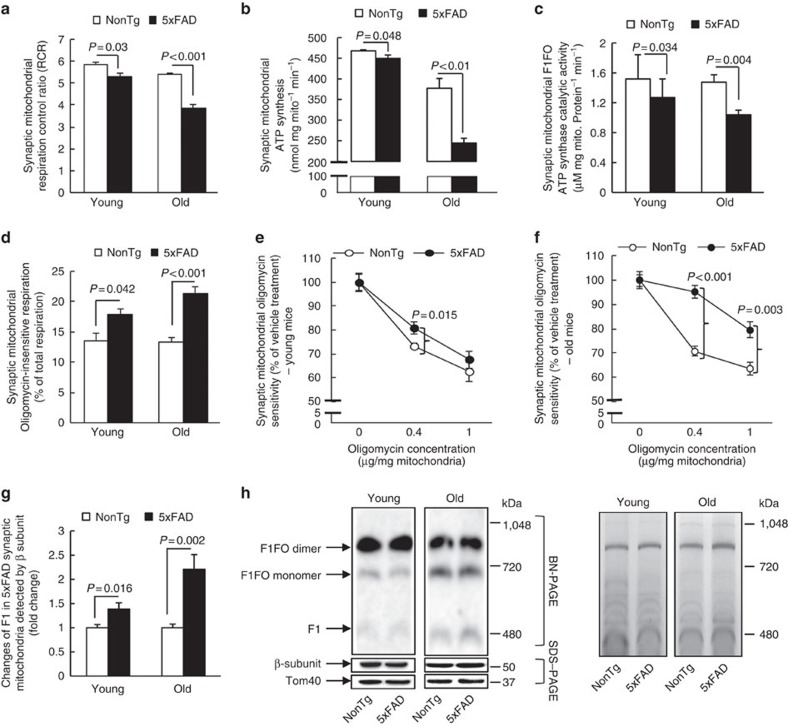
F1FO-ATP synthase deregulation in 5xFAD mouse synaptic mitochondrial. (**a**) Synaptic mitochondria from 5xFAD mice demonstrated an age-dependent decrease in their respiratory control ratio (RCR). Six nonTg and 5 5xFAD mice at 4 months old and 6 nonTg and 5 5xFAD mice at 9 months old were used. (**b**) Synaptic mitochondria from 5xFAD mice had an age-dependent decrease in ATP synthesis. Six nonTg and 6 5xFAD mice at 4 months old and those from six nonTg and seven 5xFAD mice at 9 months old were used in the experiments. (**c**) Synaptic mitochondria from 5xFAD mice demonstrated an early decrease in the F1FO-ATP synthase catalytic activity at 4 months old which was exacerbated at 9 months old. Five mice of each group at 4 months old and seven nonTg and nine 5xFAD mice at 9 months old were used in the experiment. (**d**) Age-dependent increase in oligomycin-insensitive respiration of synaptic mitochondria from 5xFAD mice. Six nonTg and five 5xFAD mice at 4 months old, and six nonTg and five 5xFAD mice at 9 months old were used in the experiments. (**e**,**f**) Decreased oligomycin sensitivity of synaptic mitochondria from 5xFAD mice at 4 (**e**) and 9 months old (**f**). All data are presented as percentage of the activity of the corresponding vehicle-treated mitochondrial fractions. Six nonTg and five 5xFAD mice at 4 months old, and seven nonTg and seven 5xFAD mice at 9 months old were used in the experiments. (**g**,**h**) Increased F1 dissociation in synaptic mitochondria from 5xFAD mice. (**g**) The analysis of free F1. (**h**) The left panel is the representative of images collected from seven nonTg and six 5xFAD mice at 4 months old, and six nonTg and six 5xFAD mice at 9 months old. F1 was determined by probing with anti-β subunit antibody and the molecular weight of the bands. The same amount of samples was used for SDS–PAGE and Tom40 and β subunit were detected to show the loading amount of mitochondrial proteins. The right panel is the coomassie blue staining before immunoblotting to indicate the loading amount of samples. Error bars represent s.e.m.

We hypothesized that if the above effects of OSCP loss and OSCP/Aβ interplay on F1FO-ATP synthase function could be extrapolated to an *in vivo* setting, 5xFAD mice would display F1FO-ATP synthase dysfunction, particularly in synaptic mitochondria, which demonstrated early and extensive OSCP reduction, as well as Aβ deposition (Supplementary Fig. 9a,b). To address this, we first measured mitochondrial OXPHOS function in 5xFAD mitochondria. Although nonsynaptic mitochondria from 5xFAD mice also demonstrated an age- and genotype-specific effect (Supplementary Fig. 10a–c), synaptic mitochondria from 5xFAD mice exhibited an early and marked decrease in the mitochondrial respiratory control ratio, ATP synthesis and F1FO-ATP synthase catalytic activity (Fig. 5a–c). In contrast, mitochondrial complex IV, whose deactivation is thought to be the major OXPHOS defect in AD^8^,^9^,^10^, exhibited only a relatively mild decrease (Supplementary Fig. 10g,h), again suggesting that F1FO-ATP synthase deregulation contributes to the extensive OXPHOS suppression in 5xFAD mice. Next, we examined F1FO complex proton-flow coupling, which reflects F1FO complex integrity^44^,^45^. Synaptic mitochondria from 5xFAD mice demonstrated a significant increase in oligomycin-insensitive respiration (that is, uncoupled electron transport, Fig. 5d). This was further supported by a F1FO-ATP synthase coupling assay which showed that 5xFAD synaptic mitochondria had markedly blunted sensitivity to oligomycin-A inhibition (Fig. 5e,f). Such changes were also seen in nonsynaptic mitochondria, but the effect size was considerably smaller (Supplementary Fig. 10d–f). These results imply that F1FO complex destabilization occurs in 5xFAD brain mitochondria. Direct evidence of F1FO complex instability in 5xFAD mitochondria was collected using BN-PAGE. Free F1 was identified by immunoblotting using specific antibodies recognizing the β subunit as previously described^42^. We found an age-dependent increase in F1 dissociation (Fig. 5g,h), confirming the destabilization of the F1FO complex in 5xFAD mitochondria. Lastly, because mPTP formation is thought to be a consequence of F1FO complex uncoupling via OSCP deregulation, we measured mPTP formation susceptibility and found that 5xFAD synaptic mitochondria exhibited a significantly increased response to Ca^2+^-induced mitochondrial swelling (Supplementary Fig. 10i), which is in agreement with our previous findings^33^. Therefore, these data show a strong correlation between OSCP deregulation and mitochondrial dysfunction in AD-relevant pathophysiological settings.

### Protection of OSCP restoration on Aβ-exposed mouse neurons

**Figure 6:**
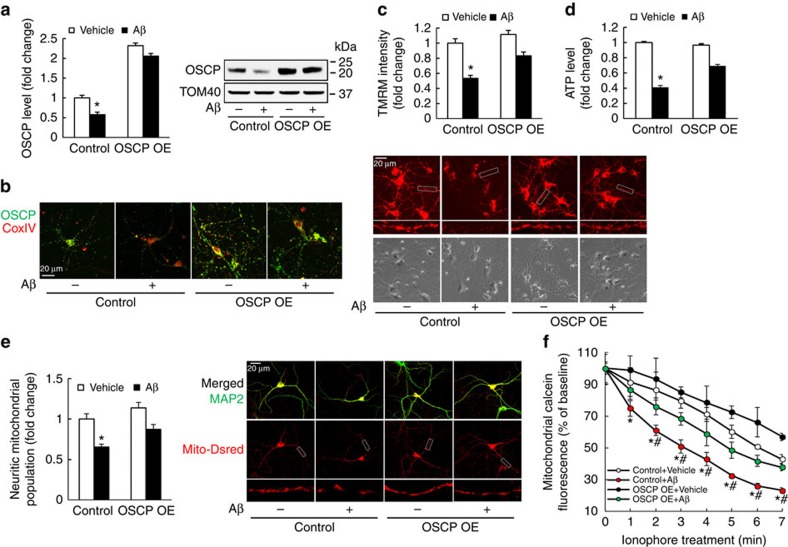
OSCP overexpression ameliorates Aβ-induced mitochondrial dysfunction in mouse neurons. Mouse cortical neurons were exposed to 500 nM oligomeric Aβ1–42 for 24 h. (**a**) Attenuated oligomeric Aβ1–42-induced OSCP reduction by OSCP overexpression. Western blot images show OSCP expression level in mouse primary neurons which is representative of seven samples in each group. (**b**) Shows immunofluorescent staining of OSCP (green) in primary cultured neurons. COXIV (red) was used to determine the localization of OSCP in mitochondria. *P<0.01 versus other groups. Scale bar, 20 μm. (**c**) OSCP overexpression protected mitochondrial membrane potential against oligomeric Aβ1–42 toxicity. The upper panel of representative images shows the TMRM staining and the lower panel shows phase contrast images. Scale bar, 20 μm. *P<0.01 versus other groups. *n*=40–64 neurons from at least three independent experiments. (**d**) OSCP overexpression protected neuron ATP reduction against oligomeric Aβ1–42 toxicity. *P<0.01 versus other groups. *n*=12 samples of each group from at least three independent experiments. (**e**) Attenuated Aβ-induced reduction in neuritic mitochondrial population by OSCP overexpression. *P<0.01 versus other groups. *n*=22–27 neurons from at least three independent experiments. The upper panel of representative images shows the merged staining of MAP2 (green, dendrite) and Mito-Dsred (red, mitochondria). Scale bar, 20 μm. The middle panel shows Mito-Dsred staining and the lowest panel shows enlarged images from the indicated areas. (**f**) Ameliorated Aβ-sensitized mPTP formation by OSCP overexpression. Ionophore (2 μM) was used to trigger mPTP formation. *P<0.05 versus vehicle-treated control and OSCP OE neurons. ^#^*P*<0.05 versus Aβ-treated OSCP OE neurons. *n*=4–6 independent experiment. Control refers to neurons infected with control lentivirus carrying backbone vector. OSCP OE refers to neurons infected with lentivirus carrying OSCP cDNA. Error bars represent s.e.m.

To further address the role of OSCP deregulation for the induction of mitochondrial dysfunction in an Aβ-rich environment we examined whether the Aβ-mediated mitochondrial dysfunction can be attenuated by OSCP restoration. Therefore, we overexpressed OSCP in mouse neurons, aiming to restore OSCP levels and to dilute the OSCP/Aβ interaction which would be expected to protect OSCP function against Aβ toxicity. The control and OSCP overexpressing (OSCP OE) neurons were exposed to a treatment with oligomeric Aβ1–42. By immunoblotting and immunostaining (Fig. 6a,b), we found that Aβ-mediated OSCP reduction in control neurons was significantly ameliorated by OSCP upregulation. Further mitochondrial functional assays showed that OSCP overexpression substantially attenuated the decreased mΔΨ, lowered ATP production and decreased neuritic mitochondrial population as well as sensitized mPTP formation in Aβ-treated neurons (Fig. 6c–f). Notably, OSCP overexpression by itself did not significantly alter the expression level of CypD (Supplementary Fig. 11). To investigate the specific protection of OSCP restoration against Aβ toxicity on neuronal mitochondria we overexpressed the F1FO-ATP synthase β-subunit, which forms the catalytic core of the F1FO-ATP synthase. We then treated the control and β-subunit-overexpressing (β-subunit OE) neurons with Aβ. Aβ treatment did not induce detectable changes in the expression levels of β-subunit in either control or β-subunit OE neurons when compared with their vehicle-treated counterparts (Supplementary Fig. 12a), which supports our findings of the unaltered β-subunit levels in AD subjects (Supplementary Fig. 1a,b) as well as in 5xFAD mice (Supplementary Fig. 3a,c). However, further experiments showed that the overexpression of β-subunit did not demonstrate significant protection against Aβ-induced mΔΨ collapse, ATP reduction or decrease in neuritic mitochondrial population, as well as mPTP activation (Supplementary Fig. 12b–e), further supporting a specific protective effect of OSCP restoration in AD-related conditions.

We used exogenous Aβ treatment to mimic the high level of Aβ over-production in AD subjects; however, it is not clear whether OSCP restoration has similar protective effects in neurons that generate endogenous Aβ. To address this question we used primary neuron cultures from 5xFAD mice. In comparison to nonTg neurons, cultured 5xFAD neurons demonstrated a significant loss of OSCP expression, which was prevented by OSCP overexpression (Supplementary Fig. 13a). Importantly, OSCP overexpression in 5xFAD neurons exhibited protective effects on mΔΨ, ATP production and neuritic mitochondrial population, as well as mPTP formation (Supplementary Fig. 13b–e) against endogenous Aβ toxicity in similar patterns as we observed in neurons treated with exogenous Aβ, confirming the protective effects of OSCP restoration against Aβ toxicity.

**Figure 7:**
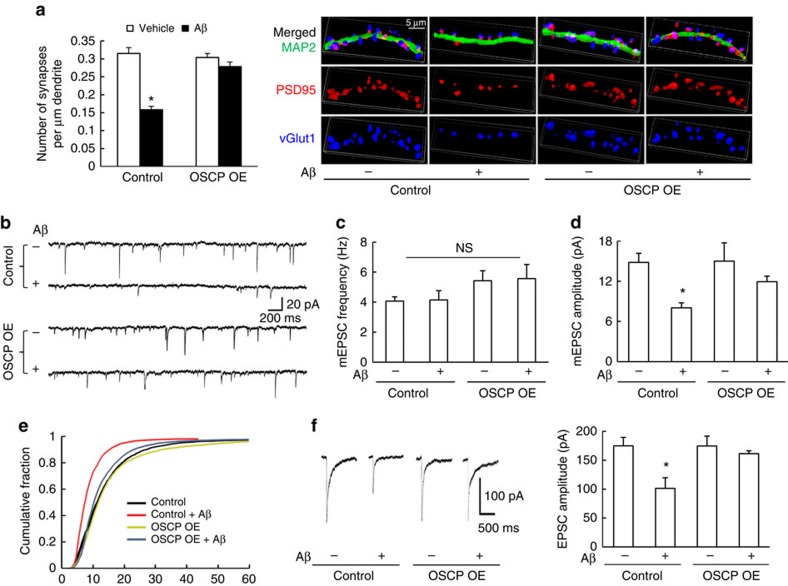
OSCP overexpression protects mouse neurons against Aβ induced synaptic dysfunction. (**a**) Attenuated Aβ-induced synaptic density reduction in OSCP OE neurons. *P<0.01 versus other groups. *n*=26–43 neurons collected from at least three independent experiments. Synapses were visualized by the staining for vGlut1 (Blue) and PSD95 (red) to identify the pre- and postsynaptic components of synapses, respectively. Neuronal dendrites were determined by MAP2 (green). Scale bar, 5 μm. (**b**–**e**) OSCP overexpression protected mEPSCs from Aβ toxicity. (**b**) Representative traces of mEPSC recordings from control and OSCP OE neurons in the presence or absence of Aβ. Scale bars represent 200 ms and 20 pA. The data were collected from 10 vehicle-treated control neurons, 8 Aβ-treated control neurons, 7 vehicle-treated OSCP OE neurons and 8 Aβ-treated OSCP OE neurons. (**c**) Quantitative analysis of mEPSC frequency. (**d**) Quantitative analysis of mEPSC amplitude. *P<0.05 versus other groups. (**e**) The cumulative fraction of mEPSC amplitude distribution. (**f**) Overexpression of OSCP prevented the reduction in amplitude of glutamate-evoked EPSCs that results from Aβ toxicity in control neurons. Scale bars represent 500 ms and 100 pA. Data were collected from seven vehicle-treated control neurons, nine Aβ-treated control neurons, five vehicle-treated OSCP OE neurons and seven Aβ-treated OSCP OE neurons. Error bars represent s.e.m.

Given the crucial role of mitochondria in sustaining synaptic transmission and plasticity^46^, and the attenuation of mitochondrial defects by OSCP overexpression, we next examined whether OSCP restoration also protects synaptic function against Aβ toxicity. Control and OSCP OE neurons were exposed to vehicle- or oligomeric Aβ1–42-treatment before the synaptic density was analysed using immunofluorescent staining of PSD95 and vGlut1. Oligomeric Aβ1–42-treated control neurons displayed a significant reduction in synaptic density in comparison to control neurons receiving vehicle treatment (Fig. 7a). In sharp contrast, the Aβ-induced synaptic loss was significantly ameliorated by OSCP overexpression (Fig. 7a). OSCP overexpression by itself did not affect baseline levels of synaptic density (Fig. 7a). In addition, OSCP overexpression protected against the reduction in mEPSC amplitude induced by Aβ toxicity (Fig. 7b,d,e). The relatively preserved mEPSC frequency of Aβ-treated neurons (Fig. 7b,c) may reflect distinct time-dependent changes in pre- and post-synaptic function during acute Aβ treatment^47^, and/or may result from presynaptic calcium accumulation due to mPTP activation^48^, which could affect asynchronous neurotransmitter release and thus mEPSC frequency^49^. Moreover, in OSCP overexpressing neurons the postsynaptic response to glutamate stimulation was indistinguishable from that in control neurons even in the presence of exogenous Aβ (Fig. 7f). Taken together, our results suggest that the restoration of OSCP protects neuronal mitochondrial and synaptic function from Aβ toxicity.

### Protection of OSCP restoration on Aβ-exposed human neurons

**Figure 8:**
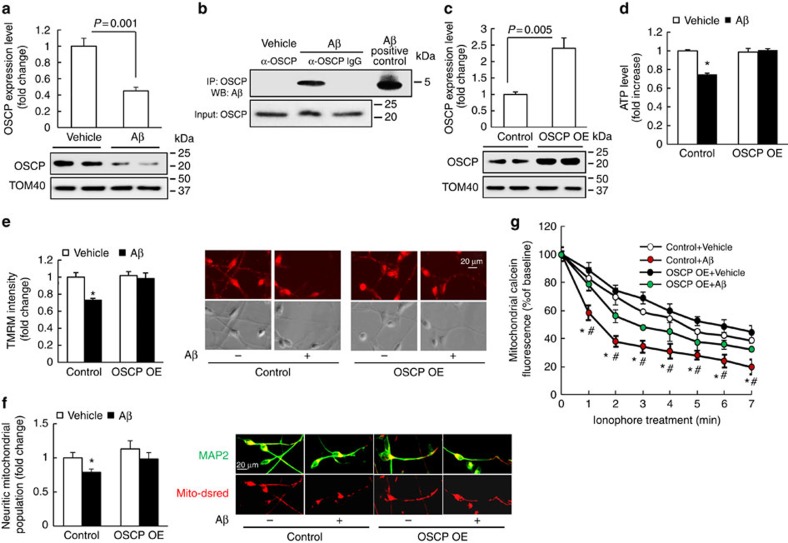
OSCP overexpression ameliorates Aβ-induced mitochondrial dysfunction in human neurons. (**a**) Human neurons were exposed to 500 nM oligomeric Aβ1–42 for 4 days, then subjected to immunoblotting detection of OSCP levels. Aβ induced a significant reduction in OSCP expression level. The lower panel shows representative immunoblotting images. Tom40 was used as the loading control. *n*=4–5 samples of each group. (**b**) Co-immunoprecipitation of OSCP and Aβ in Aβ-treated human neurons. (**c**) Significantly increased OSCP expression in the OSCP overexpressing neurons. The lower panel shows representative immunoblotting images. Tom40 was used as the loading control. *n*=4 samples of each group. (**d**) Preserved ATP production by OSCP overexpression. *P<0.01 versus other groups. *n*=6–10 samples of each group. (**e**) Preserved mitochondrial membrane potential by OSCP overexpression. *P<0.01 versus other groups. *n*=17–23 neurons from at least 3 time independent experiments. Right panels are representative images of TMRM staining (upper row) and phase contrast (lower row). Scale bar, 20 μm. (**f**) Preserved neuritic mitochondrial population by OSCP OE. *P<0.05 versus other groups. Right panels are representative images. Dendrites were determined by the staining of MAP2 (green); and mitochondria were identified by mito-Dsred (red). Scale bar, 20 μm. (**g**) Attenuated Aβ-sensitized mPTP formation by OSCP overexpression. Ionophore (2 μM) was used to trigger mPTP formation. *P<0.05 versus vehicle-treated control and OSCP OE neurons. ^#^*P*<0.05 versus Aβ-treated OSCP OE neurons. *n*=4–7 independent experiments. Error bars represent s.e.m.

Because AD is a human disease, it is of considerable interest to know whether OSCP restoration confers similar protection to mitochondria in Aβ-exposed human neurons. Therefore we derived human neurons from human neural stem cells (Supplementary Fig. 14) and treated them with oligomeric Aβ1–42. Immunobloting for OSCP levels and co-immunoprecipitation of OSCP and Aβ revealed that the OSCP loss and the formation of the OSCP/Aβ complex (Fig. 8a,b) observed in AD individuals were mirrored in Aβ-treated human neurons. Next, OSCP was overexpressed in human neurons (Fig. 8c). Control and OSCP OE human neurons were then exposed to oligomeric Aβ1–42 at 500 nM and then processed for our assays of mitochondrial function. Our results showed that Aβ-induced mitochondrial dysfunctions in ATP production, mΔΨ and neuritic mitochondrial population, as well as mPTP formation (Fig. 8d–g) were substantially reduced by OSCP overexpression in human neurons. Moreover, the overexpression of OSCP in human neurons did not have a detectable influence on baseline levels of the assayed parameters in comparison to control neurons (Fig. 8d–g), which further supports our observations in OSCP OE mouse neurons.

## Discussion

In this study, we find that OSCP loss and OSCP/Aβ interaction constitute the major OSCP alterations in the brains from AD patients and 5xFAD mice. Among the major subunits of F1FO-ATP synthase, OSCP is selectively decreased in the brains of AD subjects and 5xFAD mice. Moreover, the early decrease of OSCP expression in synaptic mitochondria from 5xFAD mice indicates that synaptic mitochondria are more vulnerable to Aβ-induced mitochondrial alterations. This reveals a novel form of synaptic mitochondrial stress in AD and supports a causative role of synaptic mitochondrial dysfunction in the development of early synaptic dysfunction in the disease^33^. Another critical finding of this study is the interaction of OSCP and Aβ. In recent years, the accumulation of Aβ in mitochondria, and particularly in synaptic mitochondria, has received considerable attention^33^. Aβ is probably transported via a translocase in the outer mitochondrial membrane^50^ and impacts mitochondrial function via multiple pathways, including the interaction of Aβ with several mitochondrial proteins such as CypD^5^, Amyloid beta-binding alcohol dehydrogenase^35^ and dynamin-like protein 1 (ref. 40). Our finding of significant interplay between OSCP and Aβ furthers our understanding of the intracellular influence of Aβ toxicity in AD. Indeed, given the extensive Aβ accumulation in mitochondria, we cannot exclude the possibility that mitochondrial Aβ may bind to other proteins embedded in the inner mitochondrial membrane or to other subunits of mitochondrial F1FO-ATP synthase, which could also be involved in compromised F1FO-ATP synthase function in AD. In view of the critical role that the β-subunit plays for the catalytic functions of F1FO-ATP synthase, we examined whether the β-subunit is a binding partner of Aβ. Our data (not shown) suggest that the β-subunit is not a likely binding partner of Aβ. Future investigation will explore whether Aβ may affect other mitochondrial proteins that contribute to mitochondrial F1FO-ATP synthase dysfunction, as well as mPTP activation in AD.

Our findings of reduced OSCP levels and the interaction of Aβ with OSCP, which impair mitochondrial function in AD-related pathological settings, implicate that Aβ toxicity induces OSCP aberrations, leading to mitochondrial F1FO-ATP synthase dysfunction in AD-sensitive brain areas. However, increasing evidence suggests that AD is a systemic disease. In addition to AD-affected brain areas, Aβ is detected in circulating blood^51^ and platelets^52^,^53^, as well as in other tissues such as intestine and kidney^54^. The systemic distribution of Aβ raises the possibility of Aβ interaction with OSCP in cells outside the nervous system, which may potentially cause mitochondrial dysfunction. Moreover, in view of the complexity of mitochondrial abnormalities in AD our findings of OSCP aberrations may only provide one mechanism for systemic mitochondrial dysfunction. Therefore, it will be important for future studies to evaluate the impact of OSCP alterations in AD on a systems level.

Suppressed mitochondrial OXPHOS is a hallmark of mitochondrial defects in AD^2^,^3^. However, the detailed mechanisms of this mitochondrial deficit in AD are not fully understood. It is well accepted that AD is accompanied by pronounced changes in the function of mitochondrial complex IV (refs 8, 9, 10). Indeed, we have found a significant decrease of complex IV activity in 5xFAD neuronal mitochondria when comparing it to the corresponding complex IV activity in nonTg mice. However, the contribution of complex IV dysfunction to ATP deficiency in AD is still controversial^11^,^12^,^13^. Given the redundant capacity of complex IV in mitochondria, it has been argued that the slight decrease in complex IV activity does not explain severe ATP deprivation in AD^11^, suggesting that other mitochondrial OXPHOS enzymes also become dysfunctional. F1FO-ATP synthase is a critical OXPHOS enzyme synthesizing ATP^18^,^19^. To date, our knowledge on the functional state of mitochondrial F1FO-ATP synthase in AD is extremely limited. Earlier studies suggested that F1FO-ATP synthase is not involved in AD because the F1FO-ATP synthase enzyme activity in the brains or platelets of AD patients^55^ appeared to be unchanged, and increased enzymatic activity was observed in platelets from probable AD subjects^56^. However, this concept has been challenged in recent years by evidence that showed alterations of this enzyme in AD-sensitive brain regions, and particularly in neurons^14^,^15^,^16^,^17^. Indeed, as we show in this study, synaptic mitochondria are more vulnerable to Aβ-induced F1FO-ATP synthase dysfunction in 5xFAD mice, whereas nonsynaptic mitochondria are relatively preserved. Further, we show that F1FO complex uncoupling is a prominent defect in 5xFAD mouse brain mitochondria. The coupling state of this enzyme in AD, however, was largely overlooked in previous studies, which failed to fully evaluate F1FO-ATP synthase deregulation in AD. Therefore, our results showing that F1FO-ATP synthase deregulation is associated with suppressed neuronal mitochondrial OXPHOS efficacy provide a novel mitochondrial mechanism of neuronal stress in AD and impact our current understanding of mitochondrial OXPHOS deficits in this disease.

The molecular identity of mPTP has long been a critical scientific issue^23^. Recently, uncoupled mitochondrial F1FO-ATP synthase was identified as the molecular basis of mPTP^23^. Specifically, the prevalent model proposes that OSCP is the binding target of CypD, which is a non-pore forming regulator of mPTP opening. The interaction of CypD with OSCP triggers the instability of the F1FO complex, thus resulting in the dissociation of F1 and eventually the formation of a nonselective leak channel within c rings^22^,^24^. These findings have suggested a key role of OSCP deregulation in the induction of mPTP. However, whether this model applies to neuronal mitochondria has not been comprehensively investigated. By downregulating OSCP in neuronal mitochondria, we found substantially sensitized mPTP formation. Importantly, this OSCP loss-associated mPTP activation is indispensable of the function of CypD, thus serving as strong evidence of the role of OSCP deregulation for inducing mPTP formation in neurons. Our *in vivo* results further link mPTP over-activation to OSCP aberrations in AD-relevant pathological settings. Sensitized mPTP formation is well documented as a key mitochondrial dysfunction in AD. Our previous studies have shown that mPTP blockade by CypD depletion protects mitochondrial and neuronal stress in a mouse model of AD, suggesting that mPTP regulation could be a therapeutic strategy for AD treatment^5^,^26^. The physiological function of mPTP has been noticed in recent years^57^,^58^,^59^. Molkentin and colleagues^57^ found increased susceptibility of heart failure in CypD-deficient mice outlining the risks associated with inhibiting the physiological functions of mPTP and CypD. Therefore, it would be preferable to develop a strategy that can reduce mPTP over-activation under pathological states like AD while preserving mPTP function under normal physiological conditions. In this study, we found that OSCP restoration reduces Aβ-induced mPTP over-activation without having a detectable influence on baseline mPTP formation. Therefore, protecting OSCP seems to be a promising avenue for the regulation of mPTP for AD treatment.

Lastly, mitochondrial dysfunction has been identified as a causative factor of synaptic failure in AD. Therefore, supporting mitochondrial function seems to be a viable strategy to protect synaptic strength and plasticity to delay the cognitive decline in AD patients^1^,^2^. We found that loss of OSCP induces synaptic dysfunction, which was paralleled by reduced mitochondrial function. Notably, the restoration of OSCP mitigated Aβ-mediated mitochondrial dysfunction and further preserved synaptic function as evidenced by our measures of synaptic density and synaptic transmission. Therefore, our results confirm the deleterious impact of mitochondrial injury on synaptic function and further highlight the therapeutic role of OSCP restoration for the protection of synaptic strength to ameliorate cognitive impairments in AD.

**Figure 9:**
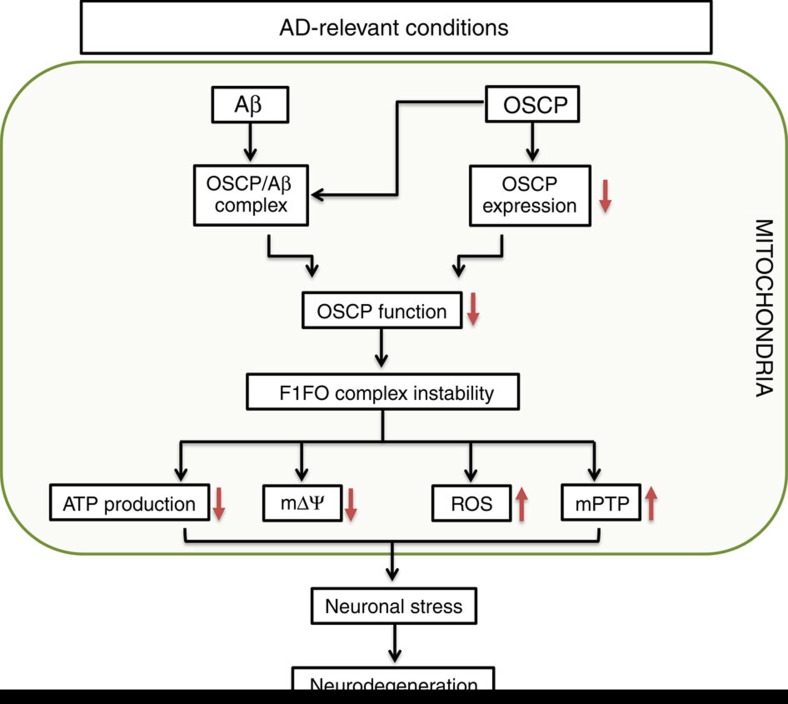
Schematic summary. With the progress of AD, brain mitochondria gradually undergo OSCP loss and Aβ accumulation in AD-relevant conditions. OSCP loss and OSCP/Aβ interaction impair OSCP function to keep F1FO complex integrity. This leads to severe mitochondrial dysfunction, including decreased ATP production, collapsed mitochondrial membrane potential, and increased ROS production and release, as well as the activation of mitochondrial permeability transition pore formation. Such mitochondrial deregulation results in dampened neuronal function and eventually neuronal death.

In summary, we have uncovered a novel mechanism of mitochondrial dysfunction mediated via OSCP disruption in an AD-relevant pathological setting. Moreover, our results indicate a role of mitochondrial F1FO-ATP synthase deregulation in the development of AD mitochondrial defects that has long been overlooked. However, other factors may also contribute to mitochondrial F1FO-ATP synthase dysfunction in AD. For example, a previous study has shown that the α-subunit interacts with extracellular domain of amyloid beta precursor protein (APP) and Aβ on neuronal surface ATPase^60^. In addition, oxidative stress disrupts ATP synthase activity^19^, and ATP synthase α-subunit O-GlcNAcylation is decreased in AD-related conditions^61^. These changes may also potentially affect mitochondrial F1FO-ATP synthase. Thus future studies need to fully explore mitochondrial F1FO-ATP synthase dysfunction in AD. Nevertheless, the most parsimonious interpretation of our findings is that F1FO-ATP synthase deregulation via OSCP links mitochondrial defects to AD (Fig. 9) and constitutes a novel target for AD therapy.

## Methods

### Mice

Animal studies were approved by the University of Texas at Dallas Institutional Animal Care and Use Committee (IACUC) and were performed in accordance with the National Institutes of Health guidelines for animal care. 5xFAD mice overexpress a human form of mAPP-bearing mutations (SwFlLon) and PSEN1 mutations (M146L and L286V) linked to familial AD. CypD-deficient mice (B6;129-*Ppif*^*tm1Jmol*^/J, mixed gender) and 5xFAD mice (B6SJL-Tg(APPSwFlLon, PSEN1*M146L*L286V) 6799Vas/Mmjax, mixed gender) were obtained from Jackson Laboratory. Mice were allocated randomly to experimental groups for the various experimental measurements based on genotyping. Four and nine months old nonTg and 5xFAD mice of mixed genders were used in the experiments. Day 0–1 nonTg, 5xFAD and CypD-deficient pups were used for primary neuron culture. The investigators performing the experiments did not select the mice allocation. The genotype of all the transgenic animals were double checked by performing PCR and/or dot blots before the experiments. Mice with wrong genotyping were excluded. The number of mice was determined by our previous data and power calculation to ensure the minimal numbers of mice as needed were used in the experiments.

### Human samples

Frozen brain samples and paraffin-embedded brain slices were requested from UT Southwestern Medical Center ADC Neuropathology Core, supported by ADC grant (AG12300) under a protocol approved by The UT Southwestern Medical Center with informed consent from all subjects and the study adhered to the Declaration of Helsinki principles.

### Mitochondria preparation

Synaptic and nonsynaptic mitochondria were isolated from tissue as previously described, brain tissues were homogenized in ice cold isolation buffer (225 mM mannitol, 75 mM sucrose, 2 mM K2PO4, 0.1% BSA, 5 mM Hepes, 1 mM EGTA (pH 7.2)) with a Dounce homogenizer (Wheaton). The resultant homogenate was centrifuged at 1,300*g* for 3 min, and the supernatant was layered on a 3 × 2-ml discontinuous gradient of 15, 23 and 40% (vol/vol) Percoll and centrifuged at 34,000*g* for 8 min (flying time) on Beckman Coulter ultracentrifuge (Optima XPN-90 Ultracentrifuge). After centrifugation, the interface between 15 and 23% (Band containing synaptosomes) was collected. Additionally, the interface between 23 and 40% (containing nonsynaptic mitochondria) was removed and collected. The fractions were then resuspended in isolation buffer containing 0.02% digitonin and incubated on ice for 5 min. The suspensions were then centrifuged at 16,500*g* for 15 min. The resulting loose pellets were washed for a second time by a centrifugation at 8,000*g* for 10 min. Pellets were collected and resuspended in isolation buffer. Percoll density gradient centrifugation was performed as described above for a second time. The interface between 23 and 40% (mitochondria released from synaptosomes) was collected and resuspended in isolation buffer to centrifuge at 16,500*g* for 15 min. The resultant pellet was resuspended in isolation buffer followed by a centrifugation at 8,000*g* for 10 min. The final synaptic mitochondrial pellet was resuspended in isolation buffer and stored on ice during experiments. Protein concentration was determined using the Bio-Rad DC protein assay (Bio-Rad Laboratories).

### ATP synthase catalytic activity assay

ATP synthase activity was measured spectrophotometrically using NADH-linked, ATP-regenerating system^62^. Briefly, mitochondria of appropriate amount were placed in ATP synthase Assay buffer (100 mM Tris-HCl (pH 7.4), 2 mM MgCl_2_, 50 mM KCl, 0.2 mM EDTA, 0.23 mM NADH and 1 mM phosphoenolpyruvate). The reaction was triggered by the addition of 0.4 M ATP-Mg and recorded on a spectrophotometer (Ultrospect 2100, Amersham Biosciences) at OD340 nm for a total of 600 s at 10-s intervals.

### ATP synthase coupling assay

Mitochondrial fractions (15 μg) were placed in ATP synthase assay buffer (100 mM Tris-HCl (pH 7.4), 2 mM MgCl_2_, 50 mM KCl, 0.2 mM EDTA, 0.23 mM NADH and 1 mM phosphoenolpyruvate) in the presence of oligomycin-A at 0, 0.4 and 1 μg oligomycin-A per mg mitochondrial protein for 15 min at room temperature. After the incubation, ATP synthase activity was measured spectrophotometrically using NADH-linked, ATP-regenerating system.

### ATP synthesis assay

Aliquots of mitochondria were analysed by using the ATP Luminescent assay kit (Abcam). Briefly, mitochondria were placed in isolation buffer (225 mM mannitol, 75 mM sucrose, 2 mM K_2_PO_4_, 0.1% BSA, 5 mM HEPES, 1 mM EGTA (pH 7.2)). Mitochondria were energized with 5 mM glutamate/malate and ATP synthesis was induced with the injection of 500 μM ADP. ATP determination was accomplished following the manufacturer’s instructions.

### Mitochondrial respiration assays

Purified mitochondria were energized by glutamate (5 mM) and malate (5 mM) and subjected to respiration assays on a Clark electrode. Oxygen consumption was triggered by the addition of ADP. The mitochondrial respiratory control ratio was defined as the ratio of State III respiration/State IV respiration. To measure oligomycin-insensitive respiration, mitochondria were energized by glutamate (5 mM) and malate (5 mM) and oxygen consumption was triggered by the addition of ADP as described above. ADP-triggered respiration was collected as total respiration. Oligomycin (5 μM) was then added and the after-oligomycin respiration was collected. The oligomycin-insensitive respiration was calculated as the percentage of after-oligomycin respiration in total respiration.

### Mitochondrial swelling assay

Mitochondria were suspended in 0.5 ml of swelling assay buffer (50 μg of mitochondrial protein, 150 mM KCl, 5 mM HEPES, 2 mM K_2_HPO_4_, 5 mM glutamate (pH 7.3)). Mitochondrial swelling was triggered by the addition of calcium (500 nmol mg^−1^ of protein). Swelling was observed by immediately and continuously recording changes at OD540 nm by using a spectrophotometer (Ultrospect 2100, Amersham Biosciences) for a total of 600 s at 10-s intervals.

### Immunoblotting analysis

Samples were prepared in sample loading buffer (50 mM Tris-HCl pH 6.8, 2% SDS, 10% glycerol, 1% β-mercaptoethanol, 12.5 mM EDTA and 0.02% bromophenol blue) and proteins were separated by SDS–PAGE (10 or 12% Bis-Tris gel; Life Technologies), and then transferred to a PVDF membrane for blotting (Bio-Rad Laboratories). After blocking in TBS buffer (20 mM Tris-HCl, 150 mM sodium chloride) containing 5% (wt/vol) nonfat dry milk for 1 h at room temperature, the membrane was then incubated and gently shaken overnight at 4 °C with primary antibodies. This was followed by incubation with the corresponding secondary antibody for 1 h at room temperature. The following antibodies were used: mouse monoclonal anti ATP5A (Life Technologies, #459240, 1:5,000), rabbit polyclonal anti ATP5B (Santa Cruz, #sc-33618,1:2,000) and mouse monoclonal anti-OSCP (Santa Cruz, #sc-365162, 1:5,000), goat polyclonal anti subunit c (Santa Cruz, #sc-132636, 1:1,000), goat polyclonal anti- subunit b (Santa Cruz, #sc-162552, 1:500), goat polyclonal anti subunit a (Proteintech, #55313-1-AP, 1:5,000), rabbit polyclonal anti-TOM40 (Santa Cruz, #sc-11414, 1:1,000), rabbit polyclonal anti-Aβ (CST, #8243, 1:5,000), mouse anti-β actin (Sigma-Aldrich, #5441, 1:10,000), mouse anti-Cyclophilin D (Millipore, #AP1035, 1:3,000), rabbit anti-VDAC (CST, #4661, 1:5,000), Goat anti-mouse IgG HRP conjugated and goat anti-rabbit IgG HRP conjugated (Life Technologies, #626520 and 656120, 1:2,000–8,000). Images were collected on Bio-Rad Chemidoc Imaging System. Image J software (National Institutes of Health) was used to analyse the scanned blots and to quantify protein signal intensity.

### Co‐immunoprecipitation

Mitochondria from cerebral cortices of transgenic mice or human subjects were suspended in buffer (500 μg ml^−1^, 50 mM Tris, 150 mM NaCl, 1 mM EDTA, protease inhibitors (Calbiochem, set V, EDTA free), 0.1% NP-40, pH 7.5) and then subjected to five freeze-thaw cycles, followed by centrifugation at 12,000*g* for 10 min at 4 °C. We immunoprecipitated the resulting supernatant with mouse antibody to OSCP (Santa Cruz, #sc-365162, 0.5 μg IgG per 100 μg protein) at 4 °C overnight, followed by an incubation with protein A/G agarose (Pierce) for 2 h at room temperature. Nonimmune IgG (0.5 μg IgG per 100 μg protein) was used as negative control. We subjected the resultant immunoprecipitant to immunoblotting with antibody to Aβ (CST, #8243, 1:4,000).

### Immunostaining analysis

For brain slices, slices were deparaffinized and rehydrated before they were subjected to antigen retrieval by boiling in citric acid buffer for 15 min. For cultured neurons, neurons were fixed in 4% paraformaldehyde for 30 min at 37 °C. After blocking (5% Goat serum, 0.3% Triton X-100, PBS), the slices were incubated with antibodies against Aβ (CST, #8243,1:1,000), COXIV (CST, #4844, 1:300), MAP2 (Sigma-Aldrich, #M4403, 1:300), OSCP (Santa Cruz, #sc-365162, 1:200) and with Nissl staining (Life Technologies, #N21479, 1:200) in combination or separately. After washing in PBS, the slices were probed with appropriate secondary antibodies including Alexa Fluor@ 488 and Alexa Fluor@ 594 (Life Technologies, 1:500). Images were collected on a Nikon confocal microscope.

### GST-OSCP pulldowns

GST pulldown was performed according to manufacturer’s protocol (Pierce). Briefly, the human OSCP cDNA (Gene Name: ATP5O. NCBI Gene ID: 539) or deleted form of OSCP cDNA was transformed into BL21 (DE3) pLysS *Escherichia coli* (Promega) using the pGEX-4t-1 plasmid (GE Healthcare). After transformation and selection a single colony was chosen for PCR to verify positive transformation. After overnight growth and induction by IPTG (Sigma-Aldrich), bacteria were pelleted and then were lysed by sonication in 1 × PBS containing 0.2 mM PMSF and 100 μg ml^−1^ lysozyme. After sonication bacterial debris was removed by centrifugation at 12,000*g* for 15 min at 4 °C. Supernatant was collected and incubated with glutathione agarose high-capacity, high-performance resin (Pierce) for 2 h. Glutathione beads were then washed and incubated overnight at 4 °C with mitochondria lysates or Aβ peptide. After washing, protein was eluted from the beads and separated by SDS–PAGE. Immunoblotting or coomassie staining was performed to visualize results.

### Protein–protein predictions

HADDOCK (high ambiguity-driven protein–protein docking)^63^ was used to predict protein–protein interaction between ATP5O (PDB# 2WSS; s chain) and amyloid beta (PDB# 1Z0Q). The modelling was performed in ambiguous interaction restraints with CPORT’S active and passive constraints. Models with *z*-scores below −1.4 were selected and amino acids on OSCP within 4 Å of amyloid beta were determined using PyMOL^64^.

### Deletion constructs

Regions of interest were determined by both HADDOCK modelling and based on literature analysis^65^,^66^. Four OSCP deletions constructs were made; OSCPΔ1–18, OSCPΔ168–190, OSCPΔ1–18; 168–190 and OSCPΔ107–121. Theses constructs were generated via PCR and inserted into a GST-bearing vector (pGEX-4t-1). The vector was subsequently transformed into BL21(DE3) pLysS *E. coli* (Promega). After transformation and selection of a single colony was chosen for PCR to verify positive transformation. Protein products were obtained via IPTG induction and purification with glutathione agarose high-capacity, high-performance resin (Pierce).

### Oligomeric Aβ preparation

Aβ1–42 peptide (GenicBio) was diluted in 1,1,1,3,3,3,-hexafluoro-2-propanol to 1 mM using a glass gas-tight Hamilton syrings with a Teflon plunger. The clear solution was then aliquoted in microcentrifuge tubes, and it was dried by vaporation in the fume hood. Peptide film was diluted in DMSO to 5 mM and sonicated for 10 min in bath sonicator. The peptide solution was resuspended in cold HAM’S F-12 to 100 μM and immediately vortexed for 30 s. The solution was then incubated at 4 °C for 24 h.

### ELISA assay for mitochondrial Aβ measurement

Aβ level in mitochondrial fractions was measured by using human Aβ1–40 and Aβ1–42 ELISA kits (Life Technologies) following the manufacturer’s instructions.

### Mouse neuron culture

Cortices or hippocampi were dissected from day 0–1 pups in cold Hank’s buffer (without Ca2+ and Mg2+), dissociated with 0.05% trysin at 37 °C for 15 min followed by 10 times trituration in ice cold neurobasal A medium. Cells were then passed through 40 μm mesh cell strainer (Corning) and centrifuged for 2 min at 200*g*. The pellet was gently resuspended in neuron culture medium (neurobasal A with 2% B27 supplement, 0.5 mM L-glutamine, 50 U ml^−1^ penicillin, and 50 μg ml^−1^ streptomycin) and plated on poly-D-lysine- (Sigma-Aldrich) coated culture plates (Corning) or Lab-Tek chamber slides (Nunc, 177445) with an appropriate density.

### Human neural stem cell culture and neural cell differentiation

Human neural stem cells (StemPro Neural Stem Cells, #A10509-01, Life Technologies) were cultured and differentiated into neurons as manufacturer’s instruction. Differentiated neurons were determined by the morphology as well as the staining of neuronal-specific marker βIII tubulin using an antibody against βIII tubulin (CST, #D71G9, 1:300) followed by a secondary antibody of Alexa Fluor@ 594 (Life Technology, #A-11037, 1:500).

### OSCP knockdown in mouse primary neurons

Lentivirus-expressing shRNA targeted to mouse OSCP was packaged with lentivirus shRNA construct (clone TRCN0000076166: 5′-CCGGGCTTCCTGAGTCCAAACCAAACTCGAGTTTGGTTTGGACTCAGGAAGCTTTTTG-3′, Sigma-Aldrich), packaging vector psPAX2 (Addgene) and envelope vector pMD2.G (Addgene). Lentivirus-expressing nontarget shRNA control (SHC002, Sigma-Aldrich) was used as a control. Mouse primary neurons were cultured for 3 days before infection with lentivirus at a multiplicity of infection (m.o.i.). of 5. The virus containing medium was removed after 2 h and fresh culture medium was replaced to continue culturing. Neurons were treated and collected for experiments after a further 7 days in culture.

### β Subunit knockdown in mouse primary neurons

Lentivirus-expressing shRNA targeted to mouse β subunit was packaged with lentivirus shRNA construct (clone TRCN0000076228: 5′-CCGGCTGCAACTGATCTCTCCATATCTCGAGATATGGAGAGATCAGTTGCAGTTTTTG-3′, Sigma-Aldrich), packaging vector psPAX2 (Addgene) and envelope vector pMD2.G (Addgene). Lentivirus-expressing nontarget shRNA control (SHC002, Sigma-Aldrich) was used as a control. Mouse primary neurons were cultured for 3 days before infection with lentivirus at an m.o.i. of 5. The virus containing medium was removed after 2 h and fresh culture medium was replaced to continue culturing. Neurons were treated and collected for experiments after a further 7 days in culture.

### OSCP overexpression in neurons

Human OSCP cDNA (Gene Name: *ATP5O*. NCBI Gene ID: 539) were inserted in to lentivirus vector with human polyubiquitin promoter-C (Addgene). Lentiviruses were packaged and applied on primary neurons similar as shRNA lentiviral vector. Oligomeric Aβ (500 nM) was used on neurons for 24 h before cell collection.

### β Subunit overexpression in neurons

Human β-subunit cDNA (Gene Name: *ATP5B*. NCBI Gene ID: 506) were inserted in to lentivirus vector with human polyubiquitin promoter-C (Addgene). Lentiviruses were packaged and applied on primary neurons similar as shRNA lentiviral vector. Oligomeric Aβ (500 nM) was used on neurons for 24 h before cell collection.

### Preparation and treatment of OSCP OE human neurons

Neural stem cells were plated on poly-ornithine (Sigma-Aldrich) and laminin (Life Technologies) coated culture plates in complete StemPro neural stem cell SFM (Life Technologies) at 2.5 × 10^4^cells per cm^2^. After 24 h, the medium was replaced with neural differentiation medium (neurobasal A medium containing 2% B27 and 0.5 mM L-glutamine; Life Technologies) for neuron differentiation. 3 days after differentiation, 10 μM fluorodeoxyuridine (Sigma-Aldrich) and 10 μM uridine (Sigma-Aldrich) were added to remove mitotic cells. After 2 days differentiation cells were infected with expressing lentivirus at an m.o.i. of 5, then were treated with 500 nM Aβ for 4 days after 7 days differentiation.

### Mitochondrial membrane potential assay

Neurons were incubated with 200 nM TMRM (Sigma-Aldrich) which is cell-permeable red colour fluorescent dye and a specific indicator of mitochondrial membrane potential. After the incubation of TMRM for 30 min in an incubator, the dye was washed by using pre-warmed neurobasal A medium and the TMRM staining on neuronal mitochondria was imaged on an inverted fluorescent microscope with on-stage incubator (Nikon). The mitochondrial membrane potential in neurites was analysed by using Nikon NIS Advanced Research software.

### Mitochondrial superoxide assay

Mitochondrial superoxide was determined by using Mitosox Red (Life Technologies)^5^,^26^,^48^. Neurons were incubated with 2 μM MitoSox Red for 30 min in an incubator followed by washing using pre-warmed Neurobasal A medium. The images of Mitosox Red staining were collected on a Nikon inverted confocal microscope with on-stage incubator. The intensity was subsequently analysed by using Nikon NIS Advanced Research software.

### Calcein AM-cobalt chloride quenching assay

Neurons were subjected to the labelling of 1 μM calcein (Life Technologies) and then incubated with 1 mM cobalt chloride (Sigma-Aldrich) to remove cytosolic calcein staining. The changes of mitochondrial calcein were recorded in the absence or presence of ionophore, A23187 (Sigma-Aldrich) which induces mitochondrial calcium overloading on a Nikon inverted fluorescent microscope with on-stage incubator (37 °C, 5% CO_2_) for 30 min. The results were subsequently analysed by using Nikon NIS Advanced Research software.

### Neuritic mitochondrial population measurement

Primary neurons were infected by lentivirus-expressing mitochondrial targeted DsRed at 2 days *in vitro* (DIV). At 9–10 DIV the neurons were fixed and stained with anti-MAP2 (Sigma-Aldrich, #M4403, 1:400) followed by goat anti-mouse IgG conjugated with Alexa 488 (Invitrogen, #A11029, 1:500). Images were collected under a Nikon confocal microscope. Neuritic segments 20 μm away from soma were used for the analysis. Neuritic mitochondrial population was calculated as the area of a neurite occupied with mitochondria.

### BN-PAGE

BN-PAGE was performed to separate mitochondrial F1FO-ATP synthase. Purified mitochondria were pelleted and resuspended in Solubilization Buffer (NativePAGE Sample Buffer (Life Technologies), Protease Inhibitor (Calbiochem, set V, EDTA free), and 1 mM PMSF with 3.33% Digitonin) and incubated on ice for 30 min. Samples were centrifuged at 12,000*g* for 10 min at 4 °C, the supernatants was recovered and mixed with G-250 Solution (¼ of detergent percentage). Samples were loaded on 3–12% NativePAGE Novex gel (Life Technologies) to separate the proteins. The intensity of coomassie staining was measured to determine the equal loading amount of samples. BN-PAGE was then transferred onto PVDF membrane with Mini-PROTEAN Tetra electrophoresis system (Bio-Rad). Proteins were subsequently fixed with 5% acetic acid and Native Mark ladder (Life Technologies) was visualized by Ponceau S staining. Blocking was performed for 1 h at RT with 5% nonfat milk. Immunoblot was performed against ATP synthase β subunit (Santa Cruz, #sc-33618, 1:5,000) or F1 (Abcam, #ab109867, 1:2,000) and OSCP (Santa Cruz, #sc-365162, 1:6,000). Images were collected on a Bio-Rad Chemidoc Imaging System. Image J software (National Institutes of Health) was used to analyse the scanned blots and to quantify protein signal intensity. For Aβ treatment experiment, purified brain mitochondria were exposed to vehicle or 5 μM oligomeric Aβ in for 30 min on ice and subsequently exposed to the incubation in the presence or absence of Ca^2+^ for 10 min. Mitochondria were then washed with mitochondria isolation buffer via centrifugation at 8,000*g* for 10 min. The pelleted mitochondria were used for BN-PAGE as described above.

### Dot blot

Dot blot was performed to confirm 5xFAD genotype. Mitochondrial or brain cortical extracts were loaded onto nitrocellulose membrane and allowed to dry. Membrane was blocked for 1 h at RT with 5% nonfat milk. Immunoblot was performed with anti-amyloid beta IgG (CST, #8243, 1:5,000). Images were taken with ChemiDoc MP System (Bio-Rad).

### Synaptic density measurement

Synaptic density of cultured neurons was measured by counting PSD95 and vGlut1-labelled clusters attaching to neuronal dendrites and presented as the number of synapses per micron of dendrite as previously described^67^. Briefly, after fixation in 4% paraformaldehyde for 30 min, neurons were blocked in 5% goat serum for 30 min at room temperature. PSD95, vGlut1 and MAP2 were labelled by rabbit anti-PSD95 (Cell Signaling, #3450, 1:200), guinea pig anti-vGlut1 (Synaptic System, #135304, 1:400) and mouse anti-MAP2 (Sigma-Aldrich, M4403, 1:400) followed by goat anti-rabbit IgG conjugated with Alexa 594 (Invitrogen, A11037, 1:500), goat anti-guinea pig IgG conjugated with Alexa 647 (Invitrogen, A21450, 1:500) and goat anti-mouse IgG conjugated with Alexa 488 (Invitrogen, A11029, 1:500), respectively. Images were collected under a Nikon confocal microscope followed by three-dimensional reconstruction by using Nikon-Elements advanced Research software^68^. The synapses were defined by colocalization of vGlut1 and PSD95. Dendritic segments between 20 and 50 μm from the soma were used for the analysis^69^.

### Electrophysiology

Whole-cell voltage-clamp recordings were obtained from cultured cells at room temperature using recording artificial cerebrospinal fluid (ACSF) containing (in mM): 126 NaCl, 2.5 KCl, 1.2 Na_2_HPO_4_, 25 Na_2_HCO_3_, 10 glucose, 2 CaCl_2_, and 1 MgCl_2_, bubbled with 95% O_2_—5% CO_2_ (refs 26, 70). Miniature excitatory postsynaptic currents (mEPSCs) were pharmacologically isolated by adding 75 μM picrotoxin and 1 μM tetrodoxin to the recording ACSF. Electrodes (2–3 MΩ open tip resistance) were filled with (in mM): 130 CsCl, 20 TEA, 10 HEPES, 2 MgCl_2_, 0.5 EGTA, 4 Na-ATP, 0.3 Na-GTP, 14 phospocreatine, and 2 QX-314, pH 7.2 (CsOH). Recordings were performed using an Axon Multiclamp 700B amplifier (Molecular Devices, Union City, CA, USA) and the data were acquired using Axograph X (Axograph Scientific, New South Wales, Australia). Cells were recorded at a holding potential of −65 mV, and access resistance of the recorded cells was monitored throughout the experiment and a <20% change was deemed acceptable. The frequency and amplitude of miniature postsynaptic currents were measured from 3 min continuous recording using MiniAnalysis (Synaptosoft, Decatur, GA, USA), with a threshold set at two times the RMS baseline noise. To directly measure changes in postsynaptic glutamate receptor function, we ‘puffed’ glutamate onto cultured hippocampal neurons during voltage-clamp recordings. Glass pipettes (1–2 μm tip) were filled with ACSF containing 1 mM L-glutamic acid and 0.5 mM Alexa Fluor 594 hydrazide. Pipettes were placed within 30 μm of the soma of the recorded neuron and glutamate was pressure ejected through a pneumatic drug ejection system (NPI Instruments, Tamm, F.R.G.), triggered by the recording software. Air pressure was set to 0.15–0.2 MPa and puff duration was between 15 and 20 ms. Fluorescence video imaging of Alexa 594 was used to ensure that glutamate puff size was comparable and that no leakage occurred between puffs. Peak amplitude was calculated from the average of at least 10 sweeps.

### Statistical analysis

Two-way ANOVA followed by Bonferroni *post hoc* analysis or Student *t*-tests wherever appropriate were used for repeated measure analysis on SPSS software (IBM software). The distribution and variance were normal and similar in all groups. *P*<0.05 was considered significant. All data were expressed as the mean±s.e.m.

## Additional information

**How to cite this article:** Beck, S. J. *et al*. Deregulation of mitochondrial F1FO-ATP synthase via OSCP in Alzheimer’s disease. *Nat. Commun.* 7:11483 doi: 10.1038/ncomms11483 (2016).

## Supplementary Material

Supplementary Information Supplementary Figures 1-14, Supplementary Table 1 and Supplementary References
