# Supplementary material for: Deregulation of mitochondrial F1FO-ATP synthase via OSCP in Alzheimer’s disease
Source: Nat Commun. 2016 May 6;7:11483. doi: 10.1038/ncomms11483 (PMC5494197; doi:10.1038/ncomms11483)
Supplement: Supplementary Information — Supplementary Figures 1-14, Supplementary Table 1 and Supplementary References [file ncomms11483-s1.pdf]

SUPPLEMENTARY INFORMATION

Supplementary figure.1

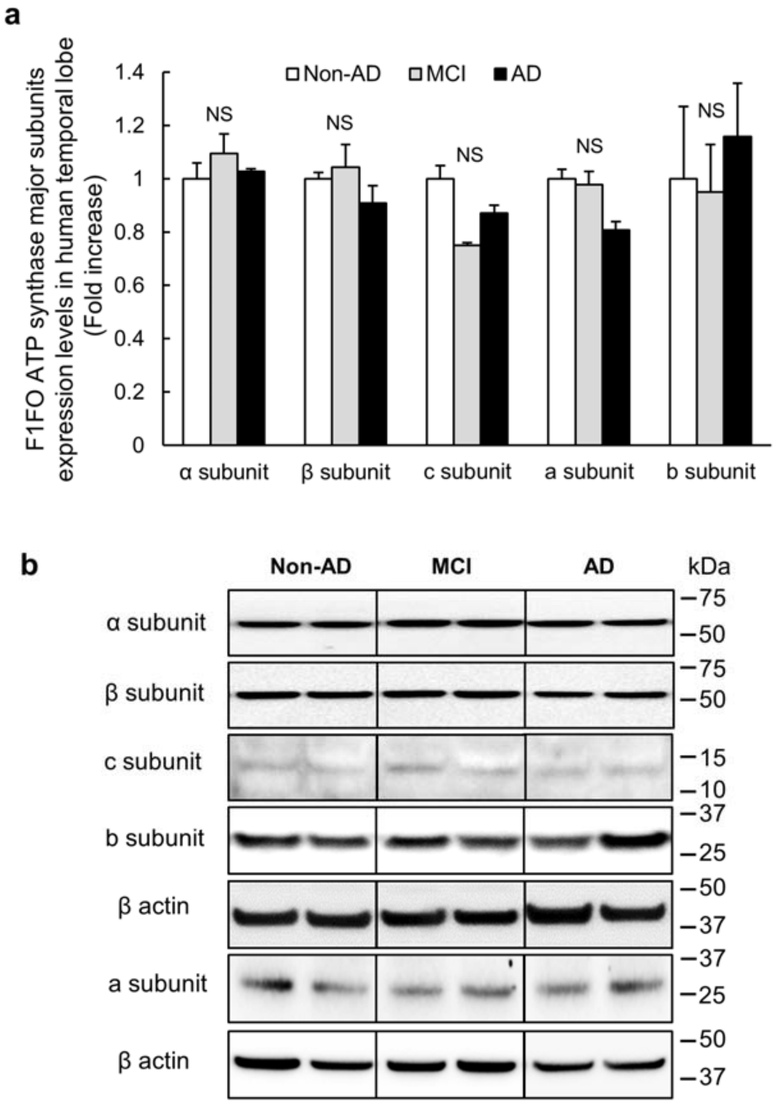

Supplementary Figure. 1 a, b, c, α and β subunits are not altered in AD cases. (a) Densitometric quantification of immunoreactive bands of major F1FO ATP synthase subunits including a, b, c, α and β in protein extracts from the temporal lobes of non-AD, MCI, and AD patients. β-actin was used to indicate the loading amount of proteins. (**b)** is representative of 6 non-AD, 6 MCI and 4 AD patients.

Supplementary Figure.2

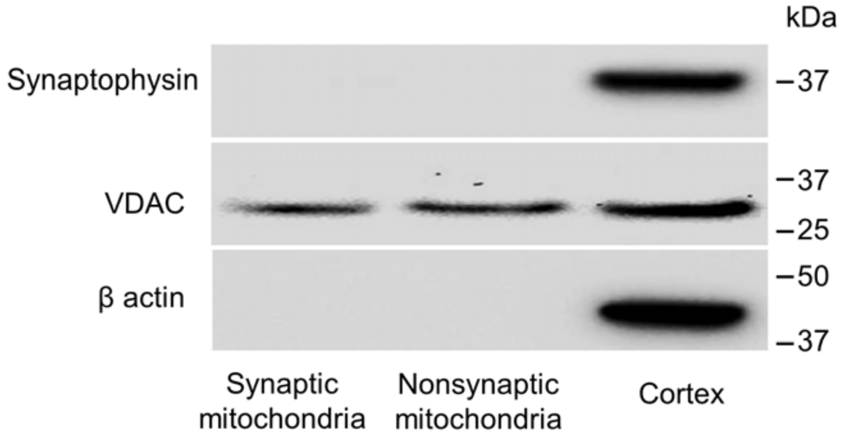

Supplementary Figure. 2 Purity of isolated mitochondria. Synaptic and nonsynaptic mitochondria were prepared following our previously published method[1]. To determine the purity of the purified mitochondria we subjected isolated mitochondria and cortex lysate to immunoblotting for the specific outer mitochondrial membrane protein voltage dependent anion channel (VDAC), synaptophysin, and β-actin. The results showed that VDAC was highly abundant, while synaptophysin and β-actin were not observed in synaptic or nonsynaptic mitochondrial fractions, suggesting no contamination by synaptic vesicles and cytosolic contents of purified mitochondria.

Supplementary Figure.3

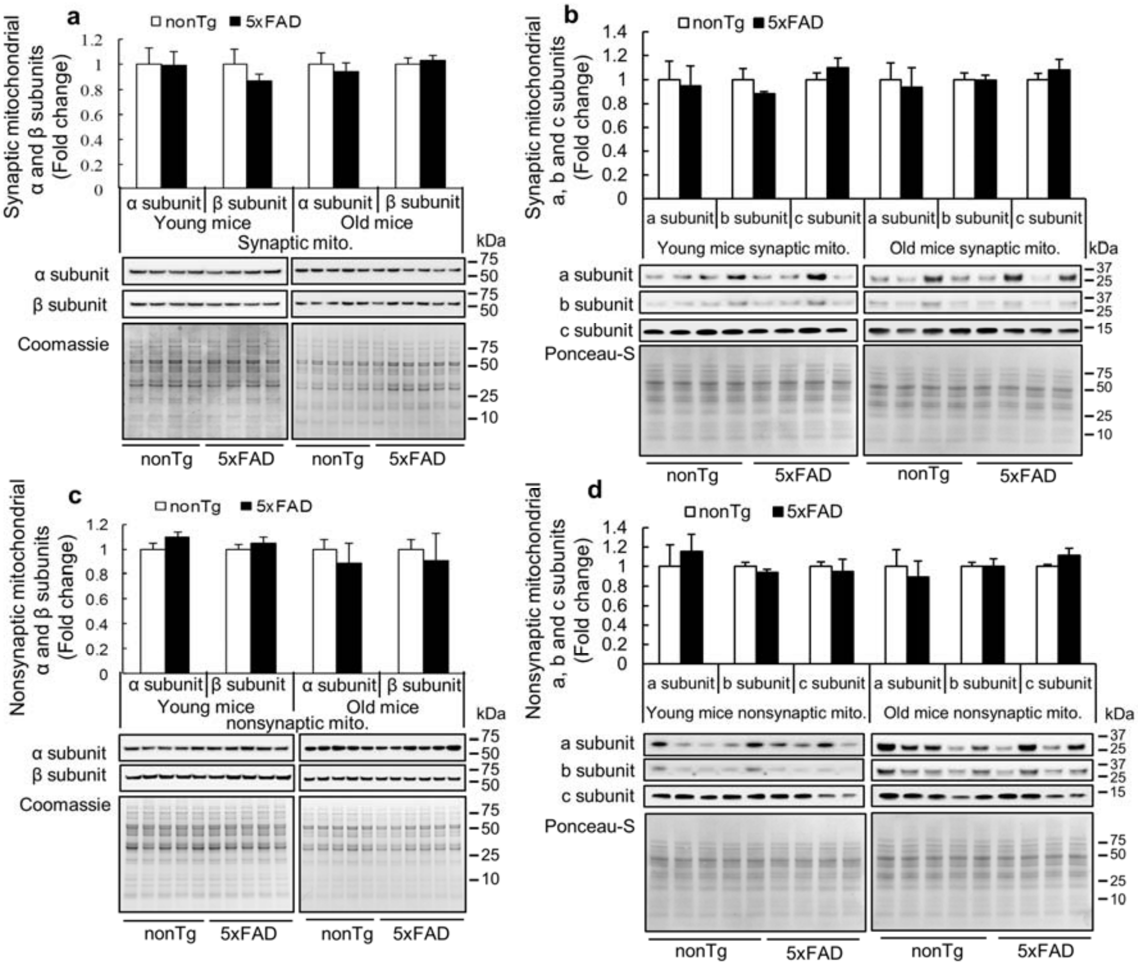

Supplementary Figure. 3 α，β，a, b and c subunits are not altered in synaptic and nonsynaptic mitochondria from 5XFAD mice. We examined the changes of α，β，a, b and c subunits in both synaptic and nonsynaptic mitochondria from 5xFAD mice. There was no significant change in any of the tested subunits in either synaptic (**a, b**) or nonsynaptic (**c, d**) mitochondria from 5xFAD mice at any given age, suggesting that OSCP is selectively decreased in 5xFAD mice. Coomassie blue or Ponceau S staining was used to demonstrate mitochondrial protein load. Five nonTg and 5 5xFAD mice at 4 months old and 5 nonTg and 6 5xFAD mice at 9 months old were used in these experiments.

Supplementary Figure.4

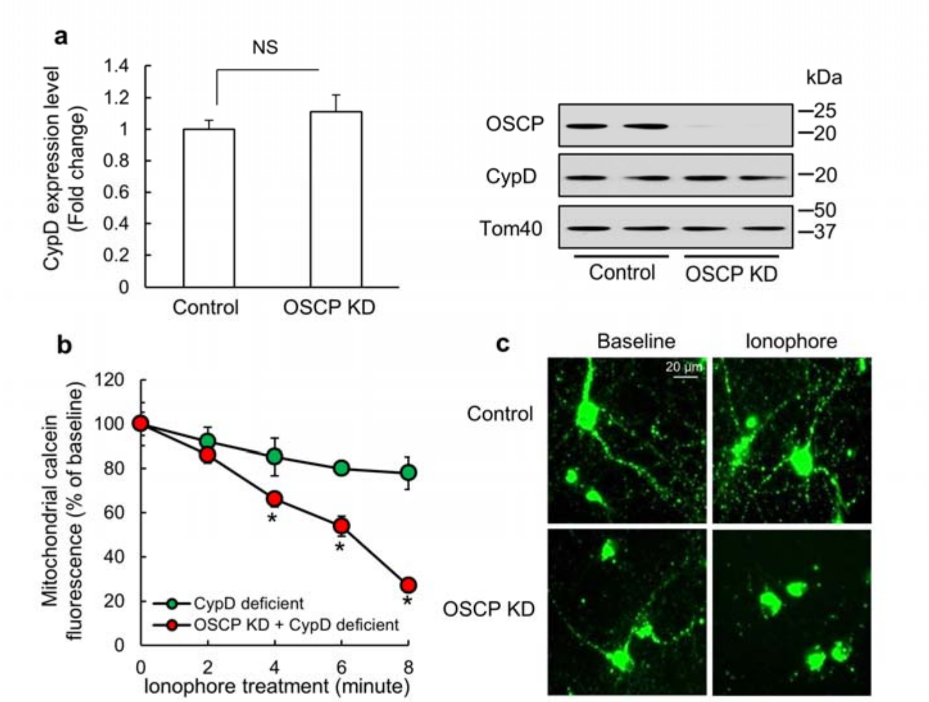

Supplementary Figure.4 OSCP loss sensitizes mPTP formation regardless of the functional status of CypD. mPTP is a pathological mitochondrial pore whose formation results in severe mitochondrial defects, including mitochondrial calcium deregulation, OXPHOS inhibition, increased ROS production, and the release of cytochrome c, eventually leading to cell death [2-5]. However, the detailed composition of mPTP remains obscure. Cyclophilin D (CypD) is so far the only known key regulator of mPTP formation, and its deficiency inhibits mPTP opening[3]. Recent studies have suggested that uncoupled F1FO ATP synthase forms the pore; and OSCP is a binding partner of CypD whose dysfunction is thought to be a triggering factor for mPTP opening[6-8]. We have observed activated mPTP formation in OSCP knock-down neurons (**Fig.2f**), which was not protected by the CypD inhibitor, Cyclosporin A (CsA) (**Fig.2f**). Further results showed that the expression level of CypD was not altered in OSCP knock-down neurons at the tested time point (**a,** Tom40 was used to show the loading amount of mitochondria). These

results suggest that CypD is indispensable for OSCP loss-induced mPTP activation. To determine whether OSCP deregulation is the key factor to sensitize mPTP formation in neurons regardless of the functional status of CypD, we examined mPTP formation in CypD-deficient neurons with lowered OSCP levels. Neurons were cultured from CypD deficient mice and subsequently exposed to either nonTarget or OSCP specific shRNA. A cobalt-quenched mitochondrial calcein assay was then performed. In the presence of ionophore to trigger mitochondrial calcium overloading[9], nonTarget shRNA-treated CypD deficient neurons demonstrated substantial protection against ionophore-induced mPTP formation (**b, c**). In contrast, the protection of CypD deficiency against ionophore induced-mPTP was dramatically reduced when OSCP was knocked down (**b, c**). *$P<0.05$ vs nonTarget shRNA-treated neurons. n=4-6 independent experiments. Taken together, the results suggest that OSCP deregulation is a major mechanism of mPTP formation in neurons.

Supplementary Figure. 5

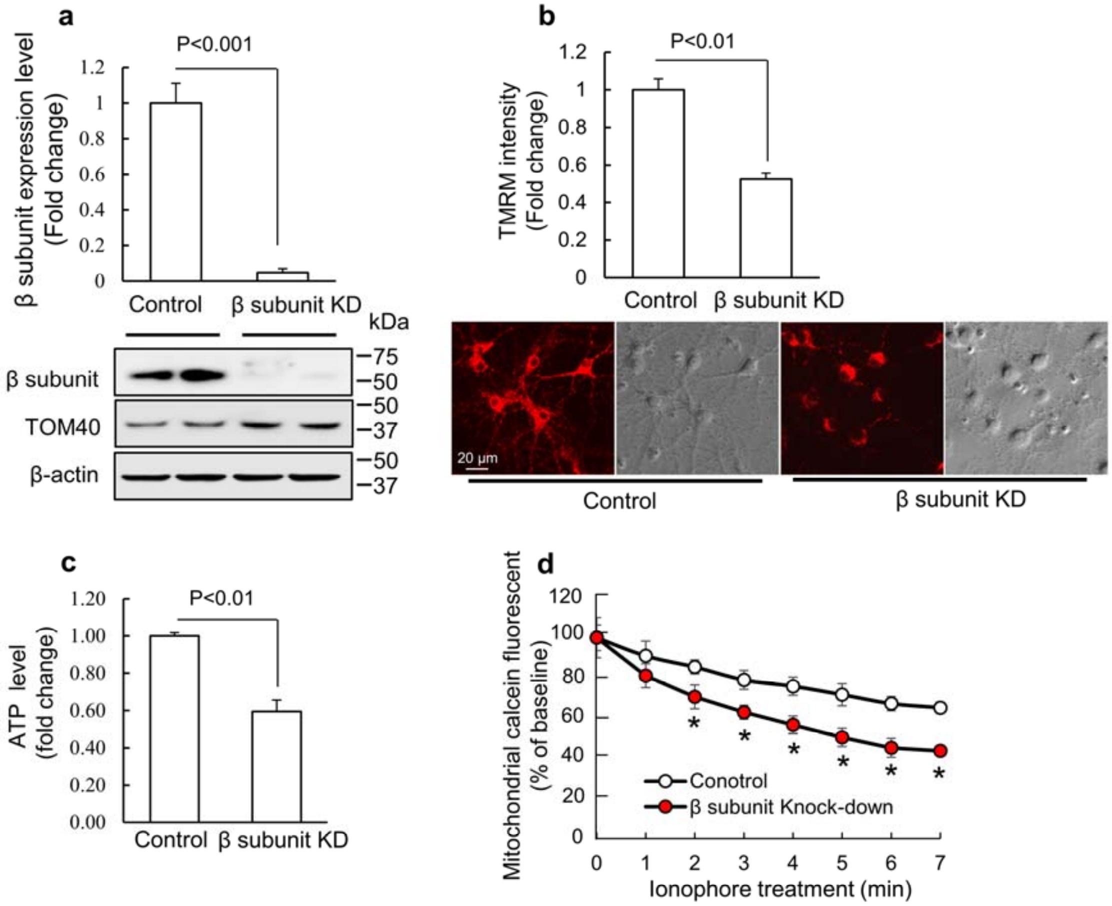

Supplementary Figure. 5 F1FO ATP synthase β-subunit down-regulation induces neuronal mitochondrial dysfunction and mPTP activation. (**a**) β subunit expression was down-regulated in primary cultured mouse neurons by using lentivirus carrying β subunit shRNA (β subunit KD) and the control neurons were treated by nonTarget shRNA. n=6 samples of each group. The lower panel shows the representative image of immunoreactive bands of β subunit. TOM40 and β actin were used as the loading control. (**b**) β subunit down-regulation induced reduction in mitochondrial membrane potential. n=14-20 neurons from at least 3 independent experiments. The lower panels are representatives of TMRM staining and phase contrast images.

Scale bar=20 μm. β subunit deficiency also induced decreased neuron ATP production (**c**). n=4 samples of each group from at least 3 independent experiments. (**d**) mPTP formation demonstrated by the drop in mitochondrial calcein intensity in the exposure of 2 μM ionophore. *P<0.01 vs β subunit KD neurons. n=5-10 independent experiments.

Supplementary Figure. 6

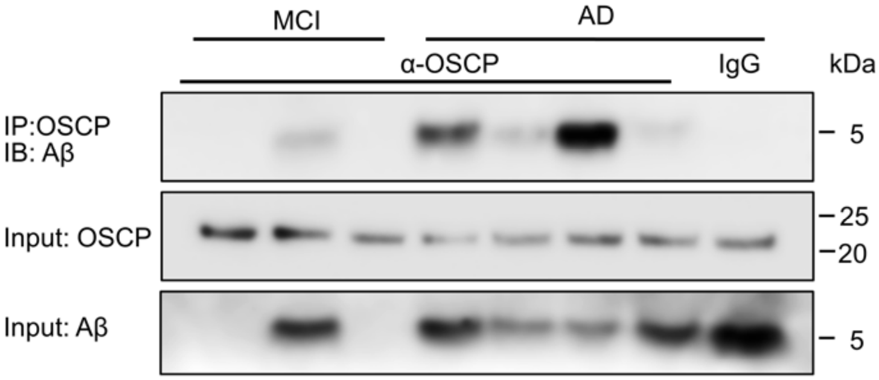

Supplementary Figure. 6 OSCP/Aβ interactions in AD and MCI subjects. Co-immunoprecipitation of OSCP and Aβ in MCI and AD patient temporal lobes. Results shown are representatives from 3 MCI and 4 AD patients. OSCP/Aβ complex was detected in one MCI subject who exhibited high level of brain Aβ; while OSCP/Aβ complex was absent in the MCI subjects with little or no brain Aβ deposition. Furthermore, the OSCP/Aβ complex was absent when anti-OSCP was replaced by non-immune IgG.

Supplementary Figure. 7

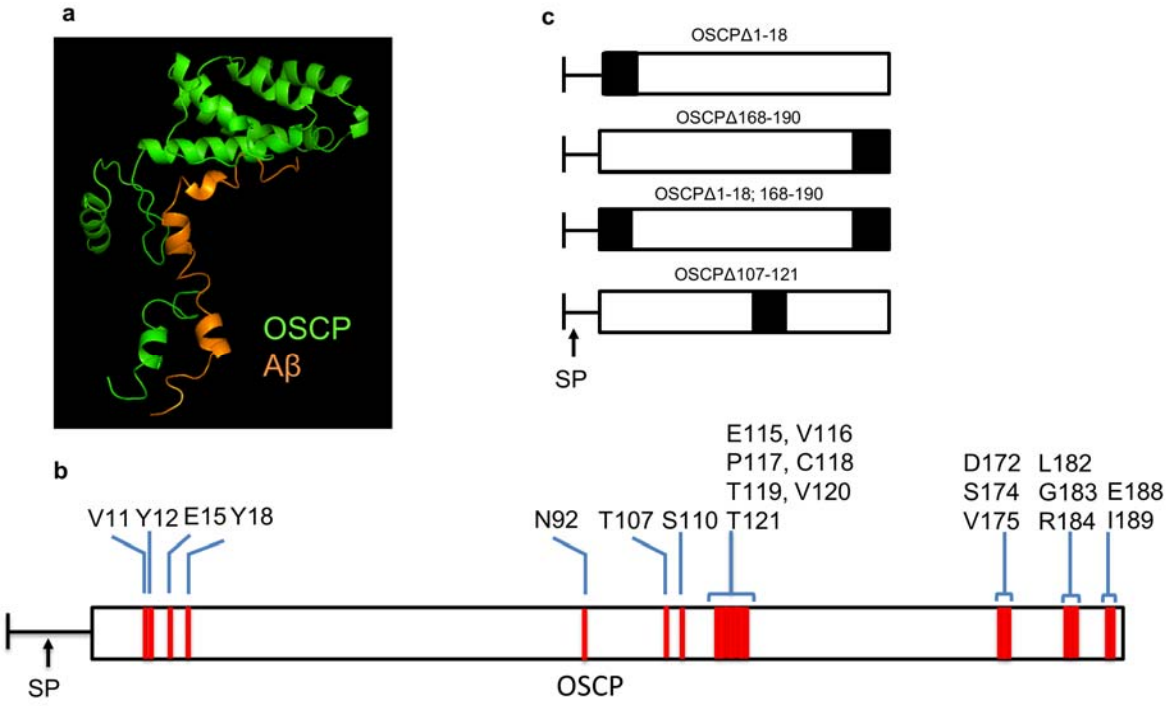

Supplementary Figure. 7 Predicted Aβ binding site on OSCP. (**a**) Aβ binding to OSCP via HADDOCK Predictions. (**b**) Predicted Aβ interacting amino acids on OSCP. (**c**) Deleted forms of OSCP used in the study.

Supplementary Figure. 8

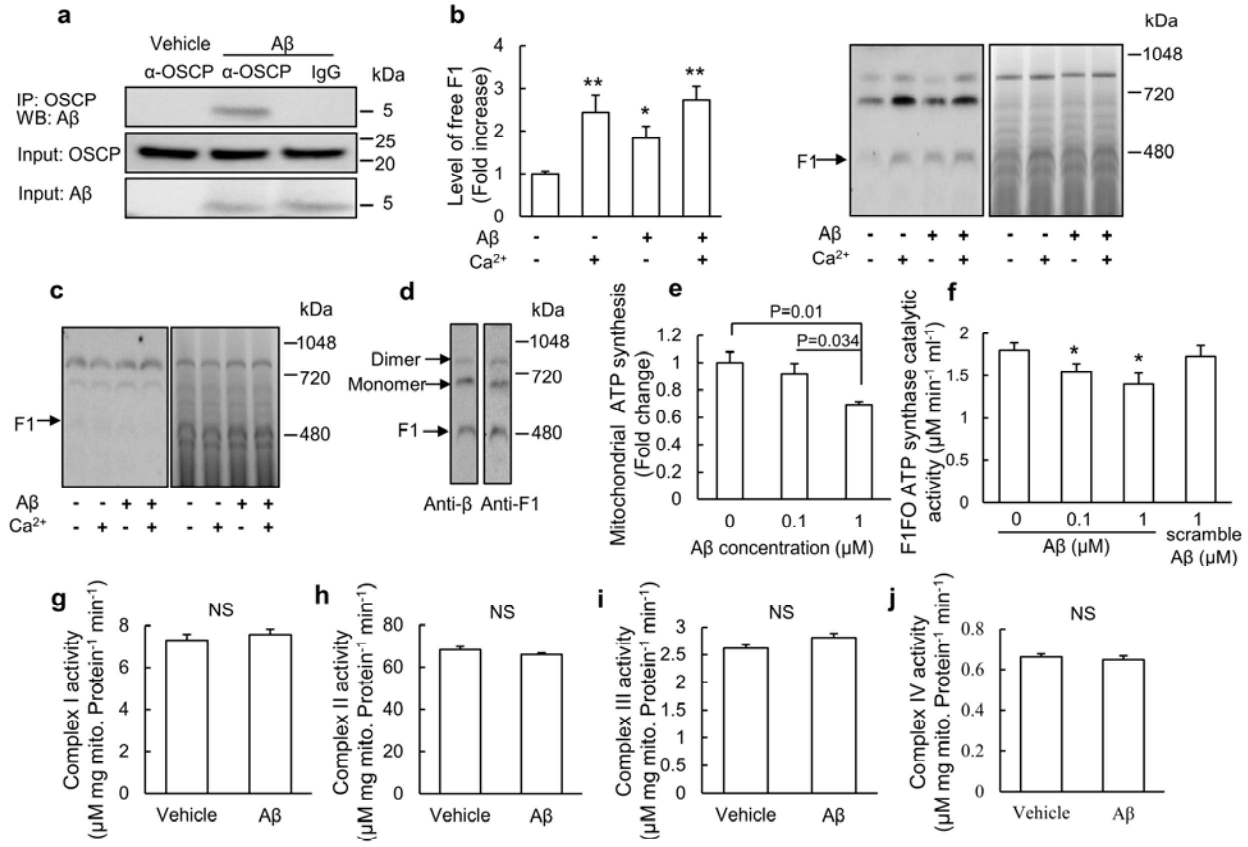

Supplementary Figure. 8 OSCP/Aβ interaction in purified mitochondria mediates F1FO ATP synthase dysfunction. We determined the physical interaction of OSCP with Aβ in AD patients as well as in 5xFAD mice. To determine the impact of OSCP/Aβ complex on mitochondrial function from OSCP loss we incubated purified brain mitochondria in vehicle or Aβ at 1μM. Co-immunoprecipitation of OSCP and Aβ was detected in Aβ-treated mitochondria. The OSCP/Aβ complex was absent when anti-OSCP was replaced by non-immune IgG (**a**). Because our *in vitro* pull-down assay indicated that Aβ inhibits OSCP function in binding F1 α and β subunits (**Fig.4f**), we also sought to see whether Aβ has a similar impact on OSCP when it is integrated within the F1FO complex. To this end, we performed BN-PAGE. F1FO dimer, monomer and F1 from digitonin-solublized mitochondria were recognized by western lots of the

β subunit as well as their molecular weights[10]. We found that Aβ treatment leads to significantly increased F1 dissociation from F1FO complex, which is similar to the effect of high level of $Ca2^+$ (**b**). *P<0.05 vs vehicle treatment. **P<0.01 vs vehicle treatment. The right panels are representatives of 5 samples from each group from 3 independent experiments using anti-β subunit. Coomassie blue staining was used to demonstrate the loaded amount of mitochondrial proteins. By performing immunoblot using anti-OSCP antibody, OSCP was detected in the F1FO complex, but was almost absent in free F1. (**c**) Representative of 5 samples of each group from 3 independent experiments using anti-OSCP and coomassie blue staining. In order to validate using anti-β subunit as a means for the identification of F1FO dimer, monomer, and F1, we used an antibody that specifically recognizes F1 (anti-F1) in parallel with the antibody against β subunit. The results showed that both antibodies labeled F1FO dimer, monomer, and F1 at the same positions (**d**). The result implies that F1 dissociation leads to loss of OSCP function by reducing the binding affinity of F1 to FO. Further mitochondrial function assays showed suppression of mitochondrial ATP synthesis by Aβ1-42 in a dose-dependent manner (**e**). ATP is the end-product of mitochondrial OXPHOS. We therefore measured the activities of mitochondrial OXPHOS enzymes and found that Aβ1-42, but not scrambled Aβ 1-42, exerts a dose-dependent inhibition of F1FO ATP synthase catalytic activity (**f**. *P<0.05 vs 0.1μM Aβ. #P<0.05 vs 1μM Aβ. n=5 of each group), while the activities of complexes I to IV were not changed at concentrations as high as 1μM Aβ (**g-j.** n=5-6 of each group), suggesting that the Aβ-induced ATP production decrease is mostly associated with destabilization of the F1FO ATP synthase under these experimental conditions.

Supplementary Figure.9

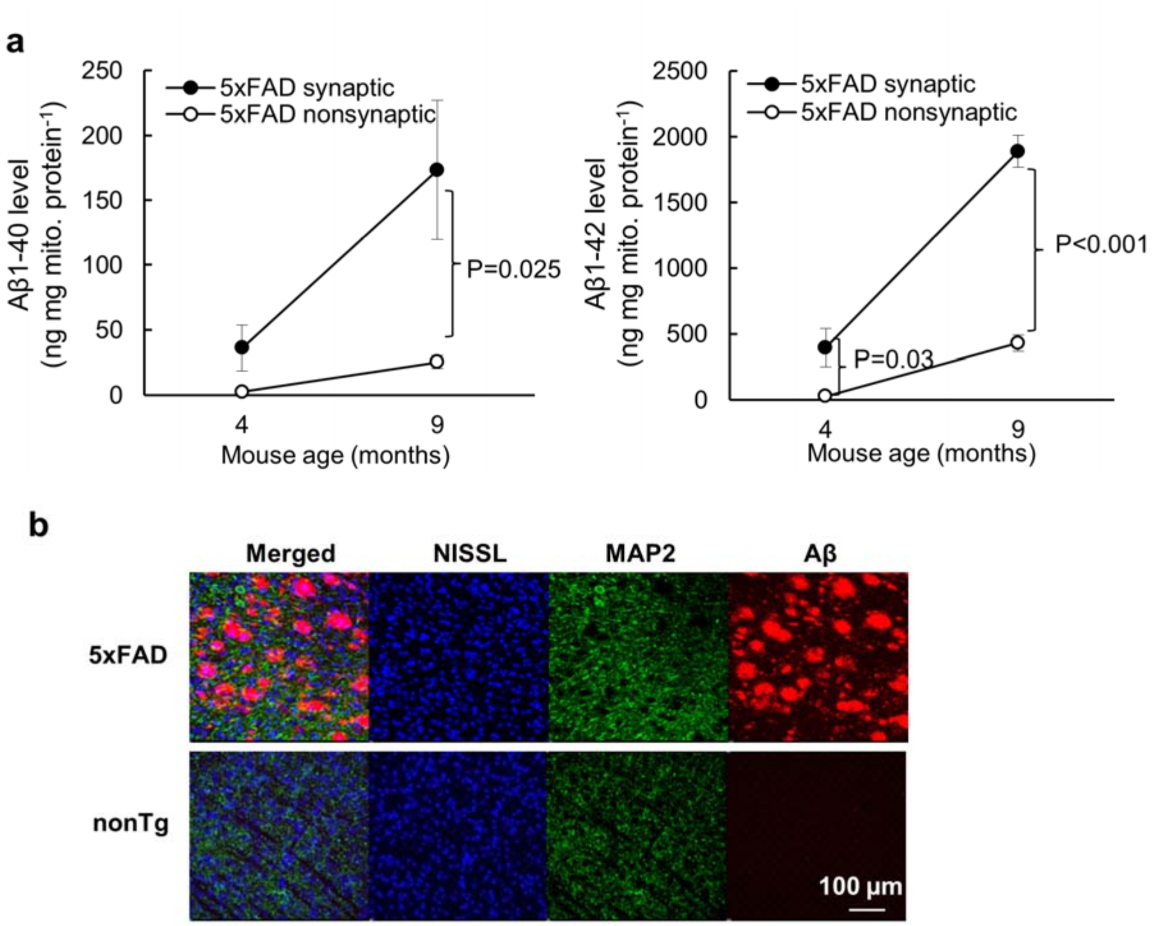

Supplementary Figure.9 Synaptic mitochondria from 5xFAD demonstrate more severe age-dependent Aβ accumulation. OSCP is a mitochondrial matrix protein. Its interaction with Aβ requires the access of intracellular Aβ into mitochondria. Previous studies have shown deposition of Aβ in brain mitochondria from AD patients and AD animal models[11-13]. However, whether Aβ accumulates in brain mitochondria from 5xFAD mice has never been tested. By using ELISA assays for the human forms of Aβ1-40 and 1-42, we determined the age-dependent increase of both forms of Aβ in synaptic and nonsynaptic mitochondria from 5xFAD mice (**a**). Notably, synaptic mitochondria demonstrated significantly higher levels of both Aβ1-40 and 1-42 than nonsynaptic mitochondria from 5xFAD mice at any given age. The results are in

agreement with our previous finding on TgmAPP mice implying that synaptic mitochondria are more susceptible to Aβ toxicity[1]. The high level of Aβ accumulation in 5xFAD mouse brain mitochondria conforms to brain Aβ production and deposition in the AD mouse model **(b)** shown by immunofluorescent staining using specific antibody against Aβ (red). NISSL and MAP2 staining were used to identify neurons. Scale bar=100 μm.

Supplementary Figure.10

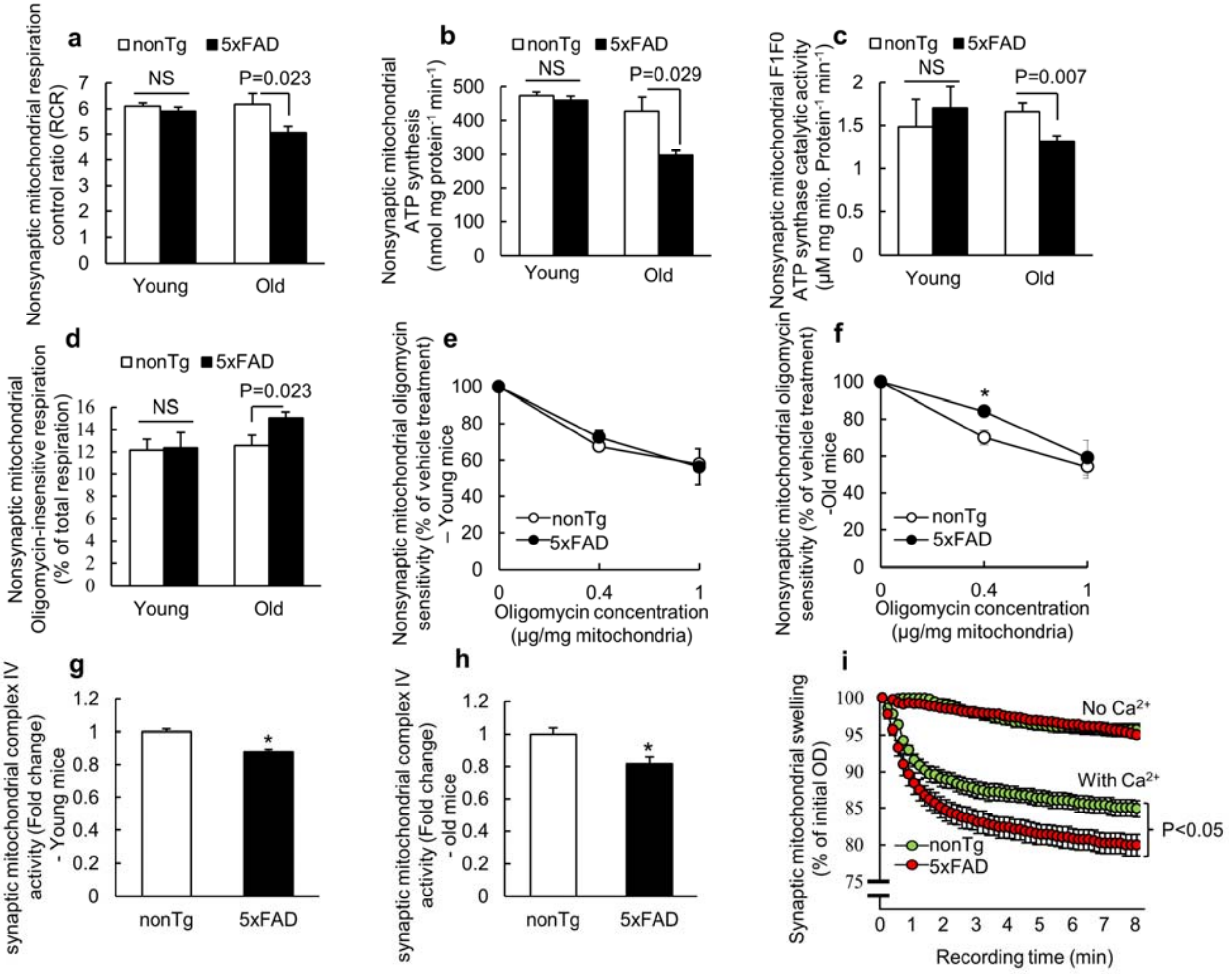

Supplementary Figure.10 5xFAD mouse mitochondria dysfunction. Mitochondrial RCR **(a)** and ATP synthesis **(b)** were not altered in nonsynaptic mitochondria from 4 months old 5xFAD mice, but significantly decreased in aged mice. Similarly, 5xFAD nonsynaptic mitochondrial F1FO ATP synthase catalytic activity was not changed in young mice, but was significantly reduced in old mice (**c**). In addition, nonsynaptic mitochondria from 5xFAD mice also demonstrated an age-dependent increase in oligomycin-insensitive respiration (**d**) with a decrease in their sensitivity to oligomycin-inhibited F1FO ATP synthase activity (**e, f**). Data

were collected from 5 mice in each group. *$P<0.05$ vs other groups. A prevailing opinion is that cytochrome c oxidase (CCO, mitochondrial complex IV) deactivation is the main mechanism underlying mitochondrial OXPHOS suppression in AD[14,15]. We found that CCO activity was significantly decreased in synaptic mitochondria from young 5xFAD mice (**g,** *$P<0.05$) and this reduction was more prominent in aged mice (**h,** *$P<0.05$). However, although statistically significant, the reduction of CCO was relatively mild and seemed not to match the substantial reduction of OXPHOS. Indeed, the role of CCO deactivation in leading to mitochondrial OXPHOS decrease in AD has been challenged recently by evidence that apoptotic neurons in AD brains have high CCO activity, and that CCO deficiency protects mitochondrial function in AD mice, implying that CCO activation is not likely the major cause. Here, our data showing severe F1FO ATP synthase deregulation in both its enzymatic activity and coupling suggest that F1FO ATP synthase dysfunction is at least one causative factor of mitochondrial OXPHOS suppression in AD. OSCP has recently been linked to mPTP formation[6-8]. We have determined that synaptic mitochondria show a significantly lowered threshold of mPTP formation as demonstrated by increased sensitivity to Ca2+-induced mitochondrial swelling which is at least partly associated with OSCP deregulation (**i**). n=4-5 mice per group.

Supplementary Figure.11

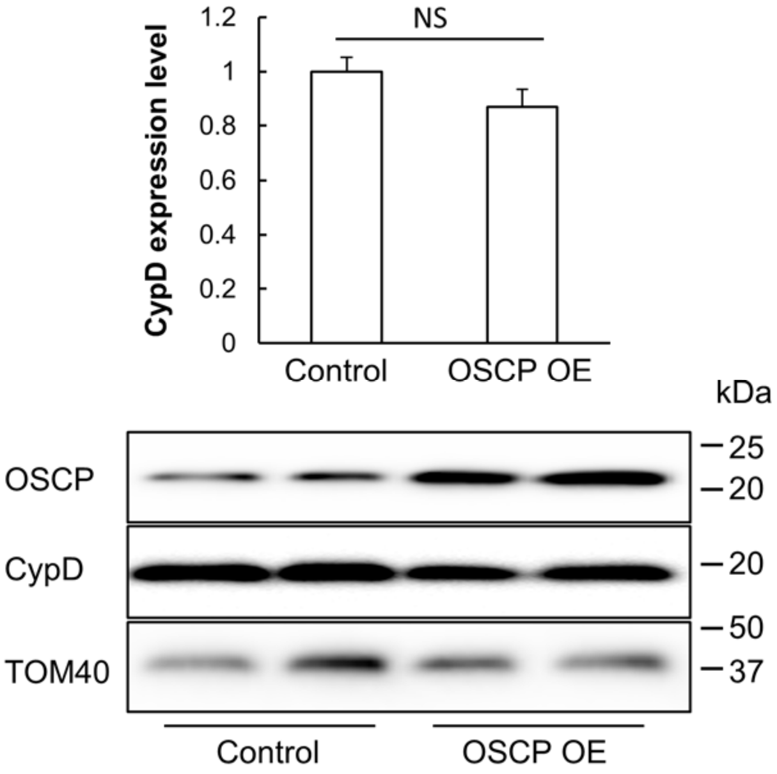

Supplementary Figure.11 OSCP overexpression does not affect CypD expression level. Overexpression of OSCP in mouse neurons induced a slight, non-significant decrease in the expression levels of CypD. The lower panel shows the representative immunoreactive bands of OSCP and CypD. TOM40 was used to determine the loading amount of mitochondrial proteins. n=4-5 samples of each group.

Supplementary Figure.12

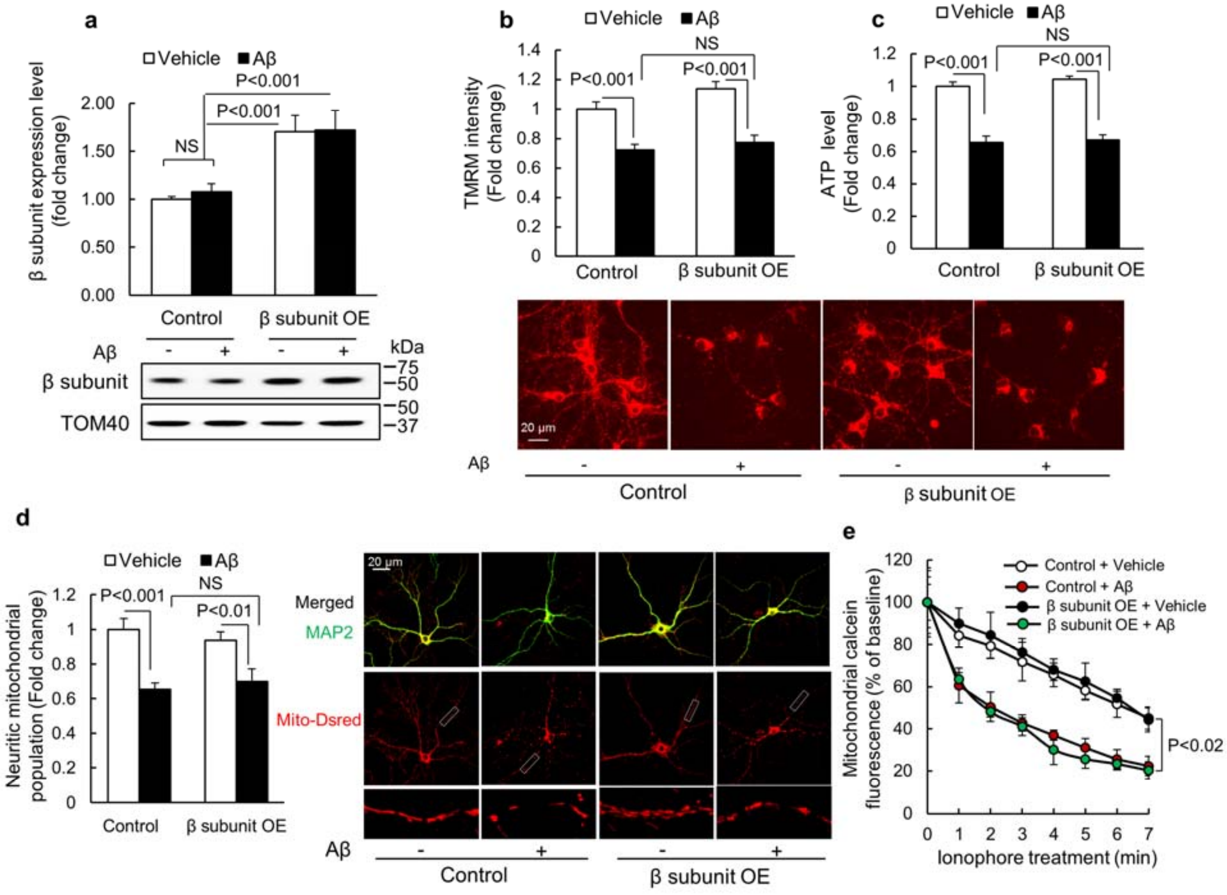

Supplementary Figure.12 β subunit overexpression has no protective effect on Aβ-mediated mitochondrial dysfunction and synaptic dysfunction in mouse neurons. Mouse neurons were exposed to 500 nM oligomeric Aβ1-42 for 24 hours. (**a**) Oligomeric Aβ1-42 had no effect on the expression levels of β subunit in neurons. n=5 samples of each group. The β subunit overexpression did not demonstrate protection on oligomeric Aβ1-42-mediated mitochondrial membrane potential collapse (**b,** n=14-25 neurons from at least 3 independent experiments), ATP reduction (**c,** n=6 samples of each group from 3 independent experiments), or decrease in neuritic mitochondrial population (**d,** n=16-26 neurons from 3 independent experiments). The lower panels of (**b**) are representative images of TMRM staining. The right panels of (**d**) are

representative images of MPA2 (green, dendrite) and Mito-Dsred (red, mitochondria) staining. Scale bar=20μm. (**e**) Aβ-sensitized mPTP formation was not altered by β subunit overexpression. 2μM ionophore was used to trigger mPTP formation. *$P<0.05$ vs vehicle-treated control and OSCP OE neurons. n=at least 3 independent experiments.

Supplementary Figure.13

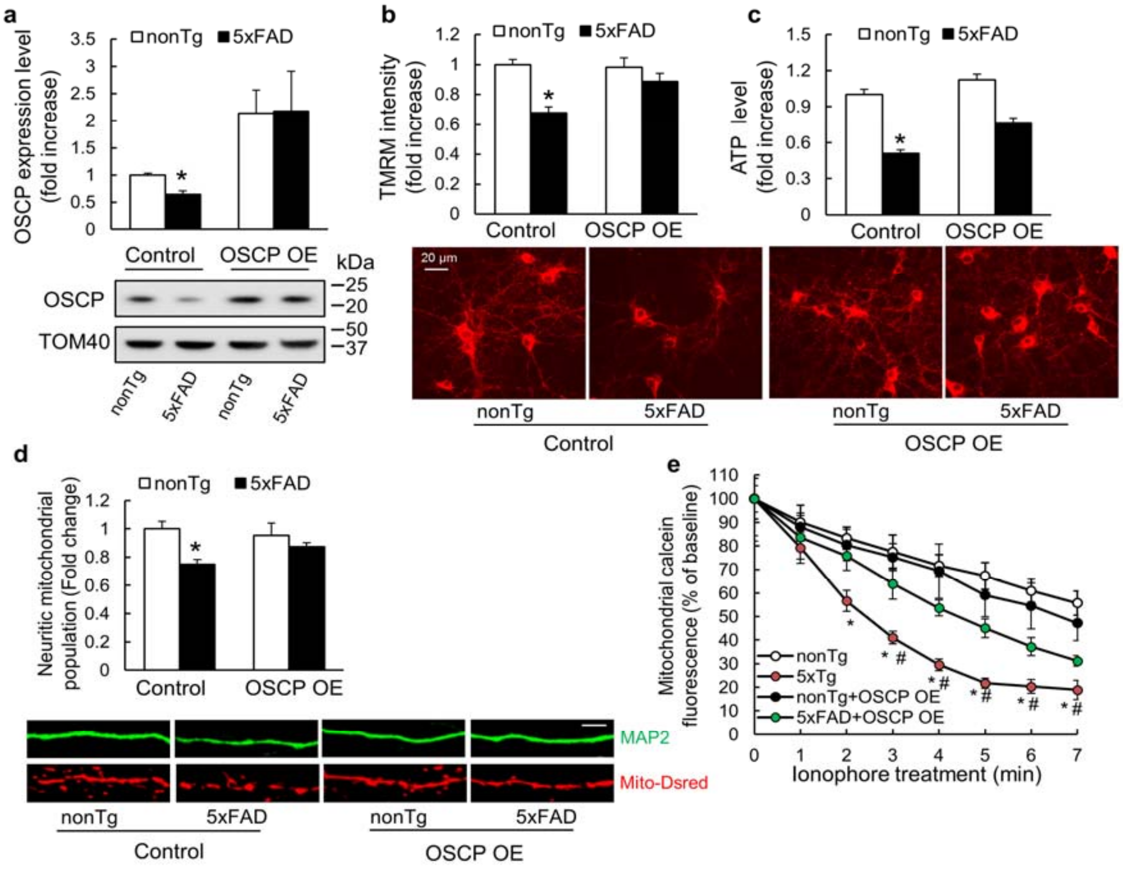

Supplementary Figure.13 OSCP overexpression mitigates endogenous Aβ-mediated mitochondrial dysfunction in 5xFAD neuron cultures. (**a**) OSCP overexpression attenuates oligomeric Aβ1-42-induced OSCP reduction in 5xFAD neurons. *P<0.05 vs other groups. n=4 samples of each group. The lower panel shows Western blot analysis of OSCP expression level. In addition, the mitochondrial membrane potential collapse (**b,** n=9-12 neurons from 3 independent experiments), ATP reduction (**c,** n=9 samples of each group from 3 independent experiments), and decrease in neuritic mitochondrial population (**d,** n=21-26 neurons from 3 independent experiments) in 5xFAD neurons were recovered by OSCP overexpression. *P<0.05 vs other groups. The lower panels of (**b**) are representative images of TMRM staining. Scale

bar=20μm. The lower panels of (**d**) are representative images of the staining of MAP2 (green, dendrite. Upper panel) and Mito-Dsred (red, mitochondria. Lower panel). Scale bar=5μm. (**e**) Sensitized mPTP formation in 5xFAD neurons was ameliorated by OSCP overexpression. 2μM ionophore was used to trigger mPTP formation. *$P<0.05$ vs nonTg and OSCP OE nonTg neurons. #$P<0.05$ vs OSCP OE 5xFAD neurons. n=5-8 independent experiments.

Supplementary Figure.14

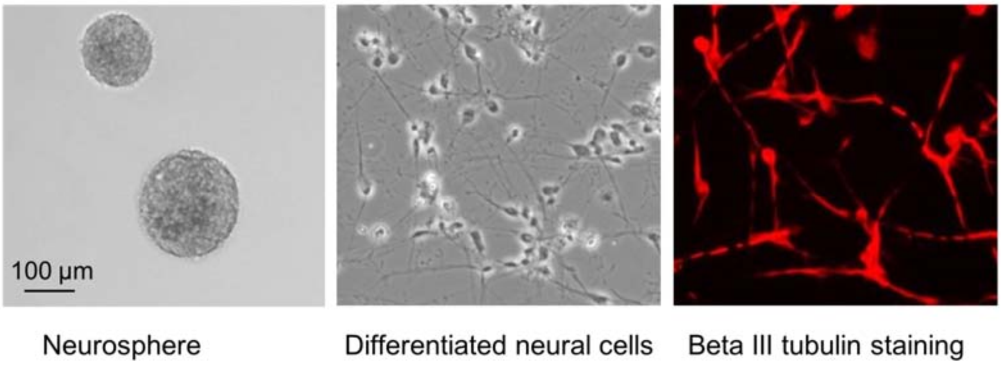

Supplementary Figure.14 Culture and differentiation of human neural stem cells. The image shows representative images of neurosphere (progenitor cells) and differentiated neural cells. The successful differentiation of neurons was determined by staining of beta III tubulin, which is a specific marker for mature neurons. The result showed that >90% of NSCs were differentiated into neurons. We used lentivirus to deliver human OSCP DNA to NSCs and subsequently differentiated the NSCs into neurons. Scale bar = 100 µm.

Supplementary Table.1: The information of the human brain tissues used in the experiments.

Clinical Dx	Case number	Gender	Age	PMI (Hr)	Braak	CERAD NP
Non-AD	36359	M	84	**24**	IV	0
Non-AD	42133	F	100	12	IV	0
Non-AD	42990	F	84	14	I	0
Non-AD	45329	M	78	21	I	0
Non-AD	39146	F	67	12	II	0
Non-AD	46202	M	77	20	II	0
**Mean ± SE**		3F/3M	81.67 ± 4.65	17.17 ± 2.19		
MCI	31606	F	84	10	III	0
MCI	34396	F	80	6	II	0
MCI	40318	M	94	26	V	Moderate
MCI	40449	F	79	7	III	Moderate
MCI	42771	M	97	25	II	Sparse
MCI	45370	M	86	14	II	Moderate
**Mean ± SE**		3F/3M	86.67 ± 3.13	14.67 ± 3.76		
AD	46090	F	75	27	VI	Frequent
AD	46121	F	74	16	VI	Frequent
AD	46991	M	62	23	V	Frequent
AD	47586	F	78	18	V	Frequent
**Mean ± SE**		3F/1M	72.25 ± 3.52	21 ± 2.48		

Non-AD, non-Alzheimer disease; MCI, Mild Cognitive Impairments; AD, Alzheimer's disease; F, female; M, male; PMI, postmortem interval; SE: standard error.

Supplementary References:

1. Du, H., *et al.* Early deficits in synaptic mitochondria in an Alzheimer's disease mouse model. *Proceedings of the National Academy of Sciences of the United States of America* **107**, 18670-18675 (2010).
2. Clarke, S.J., McStay, G.P. & Halestrap, A.P. Sanglifehrin A acts as a potent inhibitor of the mitochondrial permeability transition and reperfusion injury of the heart by binding to cyclophilin-D at a different site from cyclosporin A. *The Journal of biological chemistry* **277**, 34793-34799 (2002).
3. Baines, C.P., *et al.* Loss of cyclophilin D reveals a critical role for mitochondrial permeability transition in cell death. *Nature* **434**, 658-662 (2005).
4. Gutierrez-Aguilar, M. & Baines, C.P. Structural mechanisms of cyclophilin D-dependent control of the mitochondrial permeability transition pore. *Biochimica et biophysica acta* (2014).
5. Du, H., *et al.* Cyclophilin D deficiency attenuates mitochondrial and neuronal perturbation and ameliorates learning and memory in Alzheimer's disease. *Nature medicine* **14**, 1097-1105 (2008).
6. Alavian, K.N., *et al.* An uncoupling channel within the c-subunit ring of the F1FO ATP synthase is the mitochondrial permeability transition pore. *Proceedings of the National Academy of Sciences of the United States of America* **111**, 10580-10585 (2014).
7. Halestrap, A.P. The C Ring of the F1Fo ATP Synthase Forms the Mitochondrial Permeability Transition Pore: A Critical Appraisal. *Frontiers in oncology* **4**, 234 (2014).
8. Giorgio, V., *et al.* Dimers of mitochondrial ATP synthase form the permeability transition pore. *Proceedings of the National Academy of Sciences of the United States of America* **110**, 5887-5892 (2013).
9. Quintanilla, R.A., Jin, Y.N., von Bernhardi, R. & Johnson, G.V. Mitochondrial permeability transition pore induces mitochondria injury in Huntington disease. *Molecular neurodegeneration* **8**, 45 (2013).
10. Lemaire, C. & Dujardin, G. Preparation of respiratory chain complexes from Saccharomyces cerevisiae wild-type and mutant mitochondria : activity measurement and subunit composition analysis. *Methods in molecular biology* **432**, 65-81 (2008).
11. Lustbader, J.W., *et al.* ABAD directly links Abeta to mitochondrial toxicity in Alzheimer's disease. *Science* **304**, 448-452 (2004).
12. Takuma, K., *et al.* ABAD enhances Abeta-induced cell stress via mitochondrial dysfunction. *FASEB journal : official publication of the Federation of American Societies for Experimental Biology* **19**, 597-598 (2005).
13. Caspersen, C., *et al.* Mitochondrial Abeta: a potential focal point for neuronal metabolic dysfunction in Alzheimer's disease. *FASEB journal : official publication of the Federation of American Societies for Experimental Biology* **19**, 2040-2041 (2005).
14. Maurer, I., Zierz, S., Moller, H.J. & Jerusalem, F. Cytochrome c oxidase in Alzheimer's disease. *Neurology* **45**, 1423 (1995).
15. Mosconi, L., *et al.* Reduced mitochondria cytochrome oxidase activity in adult children of mothers with Alzheimer's disease. *Journal of Alzheimer's disease : JAD* **27**, 483-490 (2011).
